# Nickel Hydr(oxy)oxide Nanoparticles on Metallic MoS_2_ Nanosheets: A Synergistic Electrocatalyst for Hydrogen Evolution Reaction

**DOI:** 10.1002/advs.201700644

**Published:** 2017-12-04

**Authors:** Xing Zhang, Yongye Liang

**Affiliations:** ^1^ Department of Materials Science and Engineering Shenzhen Key Laboratory of Printed Organic Electronics South University of Science and Technology of China (SUSTC) Shenzhen 518055 China

**Keywords:** bifunctional mechanisms, hydrogen evolution reaction, interface engineering, metallic MoS_2_, nickel hydr(oxy)oxide

## Abstract

Molybdenum disulfide (MoS_2_)‐based materials have been recently identified as promising electrocatalysts for hydrogen evolution reaction (HER). However, little work has been done to improve the catalytic performance of MoS_2_ toward HER in alkaline electrolytes, which is more suitable for water splitting in large‐scale applications. Here, it is reported that the hybridization of 0D nickel hydr(oxy)oxide nanoparticles with 2D metallic MoS_2_ nanosheets can significantly enhance the HER activities in alkaline and neutral electrolytes. Impressively, the optimized hybrid catalyst can drive a cathodic current density of 10 mA cm^−2^ at an overpotential of ≈73 mV for HER in 1 m KOH, about 185 mV smaller than the original MoS_2_. The improved HER activity is attributed to a bifunctional mechanism adopted in these hybrid catalysts, in which nickel hydr(oxy)oxide promotes the water adsorption and dissociation to supply protons for subsequent reactions occurred on MoS_2_ to generate H_2_.

## 1 Introduction

Realization of the intriguing hydrogen economy demands efficient approaches for cost‐effective, scalable, and sustainable production of hydrogen.^1^ To address this challenge, many promising strategies such as photocatalytic or photoelectrochemical or electrocatalytic water splitting have been extensively explored.^2^, ^3^, ^4^, ^5^, ^6^ Water electrolysis such as water‐alkali electrolyzers or chlor‐alkali electrolyzers currently adopted in industrial processes is more suitable for centralized hydrogen production because the input energy is electricity which can be easily concentrated and be converted from abundant but dispersive energy sources such as wind energy, tide energy, and solar energy.^7^, ^8^, ^9^ However, unsatisfactory performance of the electrocatalysts for both the cathodic hydrogen evolution reaction (HER) and the anodic oxygen evolution reaction (OER) sets a large barrier for cost‐effective hydrogen production. Currently, platinum (Pt) or its alloys are the most efficient HER electrocatalysts with near‐zero onset potential in pH‐universal electrolytes but suffer from high‐cost and scarcity.^10^ In this regard, most efforts have been devoted to developing low‐cost and earth‐abundant alternatives for large‐scale hydrogen production in past decades.^11^, ^12^, ^13^ A variety of earth‐abundant materials including transition metal chalcogenides,^14^, ^15^, ^16^, ^17^, ^18^ phosphides,^19^, ^20^, ^21^, ^22^ carbides,^23^, ^24^ metal oxides,^25^ and metals or their alloys^26^, ^27^, ^28^, ^29^ have been identified as potential candidates. However, a large part of these electrocatalysts only exhibit noticeable activities and stabilities in acidic electrolytes but suffer from either low activity or instability in alkaline media.^30^, ^31^, ^32^ In general, HER in alkaline media shows lower kinetics and higher sensitivity to the catalyst surface structure than in acidic media.^4^, ^8^ The HER mechanism in acidic electrolytes is generally recognized as a combination of three elementary steps: (1) the Volmer step, described as hydronium (H_3_O^+^) discharge and formation of an adsorbed intermediate H_ad_^•^ on the active site (•) (H_3_O^+^ + • + e^−^ → H_ad_^•^ + H_2_O); (2) either a following Heyrovsky step (H_3_O^+^ + H_ad_^•^ + e^−^ → • + H_2_ + H_2_O) or Tafel step (2H_ad_^•^ → 2• + H_2_).^4^ In alkaline media, the HER mechanism is similar to that in acidic conditions except that H_ad_^•^ is formed from dissociation of water (H_2_O + • + e^−^ → H_ad_^•^ + OH^−^), a step with kinetic rate depending on the binding energies of H_2_O and OH_ad_ on the catalyst surface.^8^, ^9^, ^33^ Thus, the kinetic of HER in alkaline electrolytes depends on both the rate of H_ad_ combination and the rate of H_2_O dissociation.^9^ Considering that few electrocatalysts own satisfactory stabilities for OER in acidic media, water electrolyzers operating in alkaline media are more competitive than those operating in acidic media for large‐scale hydrogen production. Therefore, it is desired to develop feasible strategies to improve the activity and stability of catalysts for HER in alkaline conditions.

Due to its high catalytic activity, excellent electrochemical stability, and elemental abundance, MoS_2_ has been revealed to be a promising alternative to Pt for HER.^14^, ^15^, ^34^ Extensive efforts have been made to improve the HER activities of MoS_2_‐based catalysts in acidic media by strategies such as nanostructure engineering,^35^, ^36^, ^37^, ^38^ defect engineering,^39^, ^40^, ^41^, ^42^, ^43^ phase engineering,^44^, ^45^, ^46^, ^47^, ^48^ and heteroatom doping^49^, ^50^, ^51^ to increase the number or enhance the intrinsic activity of active sites. However, little work has been reported to improve the HER activities of MoS_2_‐based materials in alkaline media.^31^, ^52^ Recently, an Ni(OH)_2_/2H‐MoS_2_ hybrid catalyst synthesized by electrodeposition of amorphous Ni(OH)_2_ nanoparticles on 2H‐MoS_2_ nanosheets grew on carbon cloth was reported to be efficient for catalyzing HER in alkaline media.^53^ Both the electrodeposition process and all the electrochemical tests in that work were performed with a Pt foil as the counter electrode. Even though they performed preliminary density functional theory (DFT) simulation to shed light on the synergistic effect between Ni(OH)_2_ and 2H‐MoS_2_, there is no experimental evidence to exclude the effects of Ni doping and Pt contamination on the enhanced HER activities. Here, we report an interface engineering method to enhance the HER activities of metallic 1T‐MoS_2_ nanosheets in alkaline and neutral conditions by hybridization with Ni^2+^ *^δ^*O*_δ_*(OH)_2−_ *_δ_* nanoparticles. Through tuning the ratio of 1T‐MoS_2_ to Ni^2+^ *^δ^*O*_δ_*(OH)_2−_ *_δ_*, the optimized hybrid catalyst can drive a cathodic current density of 10 mA cm^−2^ at an overpotential of ≈73 mV for HER in 1 m KOH, about 185 mV less than the original 1T‐MoS_2_. Instead of nickel doping in MoS_2_ lattice, systematic experimental studies reveal that Ni^2+^ *^δ^*O*_δ_*(OH)_2−_ *_δ_* nanoparticles in the hybrid act as a cocatalyst to facilitate the adsorption and dissociation of H_2_O, supplying protons for subsequent reactions at nearby HER active sites on the metallic 1T‐MoS_2_ nanosheets.

## 2 Results and Discussion

Metallic 1T‐MoS_2_ was prepared by a hydrothermal method according to the literature.^47^ Scanning electron microscopy (SEM) image and transmission electron microscopy (TEM) images reveal an ultrathin nanosheet structure with thickness of several nanometers and lateral size of over 100 nm of the prepared MoS_2_ (Figure S1, Supporting Information). The X‐ray diffraction (XRD) pattern of the prepared MoS_2_ is significantly different from that of commercial 2H‐MoS_2_, showing new (002) and second‐order (004) diffraction peaks of MoS_2_ at 9.4° and 18.8°, respectively (Figure S2a, Supporting Information). The lattice spacing of the (002) plane is calculated to be 0.94 nm according to the Bragg equation, consistent with the measured interlayer distance from the TEM image (Figure S1d, Supporting Information). The enlargement of interlayer spacing of MoS_2_ indirectly suggested the metallic 1T phase of the prepared MoS_2_.^54^, ^55^ The Raman spectrum of the synthesized MoS_2_ is also distinct from the commercial 2H‐MoS_2_, showing additional peaks at 146 cm^−1^ (*J*_1_), 233 cm^−1^ (*J*_2_), and 334 cm^−1^ (*J*_3_) which are attributed to the phonon modes in 1T‐MoS_2_ superlattice structure (Figure S2b, Supporting Information).^47^, ^56^ The strong E_1g_ band peaked at 281 cm^−1^ along with the weak E^1^_2g_ band peaked at 375 cm^−1^ confirms the dominant octahedral coordination of Mo in 1T‐MoS_2_.^57^ The peaks at 112, 123, and 195 cm^−1^ are also observed in the Raman spectra of 1T‐MoS_2_ in some reported literatures while the attributes of these peaks are not clear so far.^47^, ^55^, ^57^ X‐ray photoelectron spectroscopy (XPS) was employed to investigate composition and chemical state of the synthesized MoS_2_. It is found that both the Mo 3d and S 2p spectra of the synthesized MoS_2_ shift to lower binding energies by ≈1 eV with respect to the corresponding peaks in 2H‐MoS_2_ (Figure S2c,d, Supporting Information), consistent with the previously observed relaxation energy of 1 eV for 1T‐MoS_2_ versus 2H‐MoS_2_.^47^, ^55^ Thus, all the XRD, Raman, and XPS results suggest the successful preparation of 1T‐MoS_2_ in this work.

**Figure 1:**
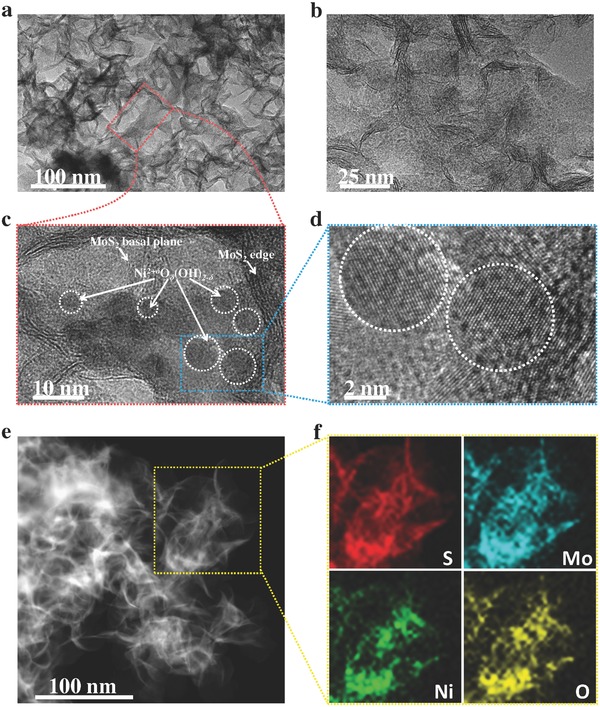
Morphology and structure characterizations of the 1T‐MoS_2_/Ni^2+^ *^δ^*O*_δ_*(OH)_2−_ *_δ_* (1:1) hybrid. a,b) Low‐magnification TEM images. c) High‐resolution TEM image of marked region in (a). d) Magnified TEM image of the marked region in (c), showing the crystal lattice of Ni^2+^ *^δ^*O*_δ_*(OH)_2−_ *_δ_* and MoS_2_. e) HAADF‐STEM image. f) Elemental mappings of the marked region in (e).

The synthesis of 1T‐MoS_2_/Ni^2+^ *^δ^*O*_δ_*(OH)_2−_ *_δ_* hybrids was performed by in situ growth of Ni^2+^ *^δ^*O*_δ_*(OH)_2−_ *_δ_* on the surface of 1T‐MoS_2_ through the reaction between NiCl_2_ and NH_4_HCO_3_ in 1T‐MoS_2_ ethanol suspension (see details in the Experimental Section). The mass ratio of 1T‐MoS_2_: Ni^2+^ *^δ^*O*_δ_*(OH)_2−_ *_δ_* can be easily tuned by controlling the input amount of NiCl_2_ in the synthetic process. For convenience, the 1T‐MoS_2_/Ni^2+^ *^δ^*O*_δ_*(OH)_2−_ *_δ_* hybrids with different mass ratio of 1T‐MoS_2_: Ni^2+^ *^δ^*O*_δ_*(OH)_2−_ *_δ_* will be thereafter defined as 1T‐MoS_2_/Ni^2+^ *^δ^*O*_δ_*(OH)_2−_ *_δ_* ([1T‐MoS_2_]_mass_: [Ni^2+^ *^δ^*O*_δ_*(OH)_2−_ *_δ_*]_mass_). The TEM images of 1T‐MoS_2_/Ni^2+^ *^δ^*O*_δ_*(OH)_2−_ *_δ_* (1:1) hybrid show its 2D nanosheet structure with many tiny nanoparticles attached on the nanosheets (**Figure** **1**a,b). The XRD pattern of the 1T‐MoS_2_/Ni^2+^ *^δ^*O*_δ_*(OH)_2−_ *_δ_* (1:1) hybrid only shows the characteristic diffraction peaks of 1T‐MoS_2_, and no characteristic peaks of the crystalline nickel species are observed, similar to the free growing Ni^2+^ *^δ^*O*_δ_*(OH)_2−_ *_δ_* (Figure S3, Supporting Information). However, high‐resolution TEM images show many crystalline nanoparticles with size around 5 nm well distributed on the basal planes and edges of the 1T‐MoS_2_ nanosheets (Figure 1c,d). The crystalline nanoparticles in either 1T‐MoS_2_/Ni^2+^ *^δ^*O*_δ_*(OH)_2−_ *_δ_* hybrid or the free growing Ni^2+^ *^δ^*O*_δ_*(OH)_2−_ *_δ_* are revealed to exist in the form of γ‐NiOOH phase in view of their fast Fourier transformation (FFT) patterns, which can be well indexed by the standard diffraction pattern of γ‐NiOOH (JCPDS No. 6–75) (Figure S4, Supporting Information). Raman‐shift peak at 480 cm^−1^ appears in both the hybrid and the free growing Ni^2+^ *^δ^*O*_δ_*(OH)_2−_ *_δ_*, which could be attributed to the O—Ni—O bending mode in γ‐NiOOH (Figure S5, Supporting Information).^58^, ^59^ XPS spectra of 1T‐MoS_2_/Ni^2+^ *^δ^*O*_δ_*(OH)_2−_ *_δ_* hybrid show no noticeable changes of the binding energy of Mo 3d, S 2p, and Ni 2p compared to those of the pristine 1T‐MoS_2_ and the free growing Ni^2+^ *^δ^*O*_δ_*(OH)_2−_ *_δ_* (Figures S6 and S7, Supporting Information), suggesting no obvious electronic interactions between 1T‐MoS_2_ and Ni^2+^ *^δ^*O*_δ_*(OH)_2−_ *_δ_* in the hybrid. The XRD, Raman, and XPS results of the 1T‐MoS_2_/Ni^2+^ *^δ^*O*_δ_*(OH)_2−_ *_δ_* hybrid suggest the maintenance of the metallic 1T phase of the MoS_2_ in the hybrid. Generally, Ni 2p XPS spectra in Ni(OH)_2_ and NiOOH are hardly distinguishable, while O 1s XPS spectra can give some information on the chemical state of Ni species.^60^, ^61^ The O 1s spectrum of the free growing Ni^2+^ *^δ^*O*_δ_*(OH)_2−_ *_δ_* can be deconvoluted into two peaks at 529.6 and 531.0 eV, corresponding to Ni—O and Ni—O—H species, respectively (Figure S6, Supporting Information).^60^, ^61^ The integrated area of Ni—O—H peak is much larger than that of Ni—O peak, suggesting the coexistence of Ni(OH)_2_ and NiOOH in Ni^2+^ *^δ^*O*_δ_*(OH)_2−_ *_δ_*.^61^ This is consistent with the TEM data, which show many amorphous nanoparticles and crystalline γ‐NiOOH nanoparticles in the free growing Ni^2+^ *^δ^*O*_δ_*(OH)_2−_ *_δ_* (Figure S4, Supporting Information). In combination with the TEM, Raman, and XPS data, it suggests that the free growing Ni^2+^ *^δ^*O*_δ_*(OH)_2−_ *_δ_* is mainly composed of amorphous Ni(OH)_2_ and crystalline γ‐NiOOH nanoparticles. The XRD, XPS, and Raman results of the 1T‐MoS_2_/Ni^2+^ *^δ^*O*_δ_*(OH)_2−_ *_δ_* hybrid suggest the nature of Ni^2+^ *^δ^*O*_δ_*(OH)_2−_ *_δ_* in the hybrid is similar to that of the free growing Ni^2+^ *^δ^*O*_δ_*(OH)_2−_ *_δ_*. However, the amorphous Ni(OH)_2_ nanoparticles are difficult to be distinguished in the TEM images of the hybrid due to the strong background of the crystalline 1T‐MoS_2_ which results in weak imaging contrast. The specific content ratio of Ni(OH)_2_: NiOOH in the hybrid could not be accurately determined. Notably, previous work has experimentally demonstrated that Ni in nickel hydr(oxy)oxide exhibits different average valence state at different potentials and is converted to Ni^2+^ state in the form of Ni(OH)_2_ under the HER potential region.^9^ In view of the similar molar weight and the easy and reversible transformation between Ni(OH)_2_ and NiOOH, it is conservative to define the nickel hydr(oxy)oxide by a nonstoichiometric formula of Ni^2+^ *^δ^*O*_δ_*(OH)_2−_ *_δ_* (0 ≤ δ ≤ 1) in the context.^9^, ^59^ The ratio variation between Ni(OH)_2_ and NiOOH in the as‐prepared hybrid will not affect the HER performance. High‐angle annular dark‐field scanning TEM (HAADF‐STEM) image of the 1T‐MoS_2_/Ni^2+^ *^δ^*O*_δ_*(OH)_2−_ *_δ_* (1:1) hybrid clearly reveals the ultrathin nanosheet structure of the hybrid inheriting from the pristine 1T‐MoS_2_ (Figure 1e). Energy‐dispersive X‐ray spectroscopy (EDS) elemental mapping results demonstrate the uniform hybridization between Ni^2+^ *^δ^*O*_δ_*(OH)_2−_ *_δ_* nanoparticles and 1T‐MoS_2_ nanosheets (Figure 1f).

**Figure 2:**
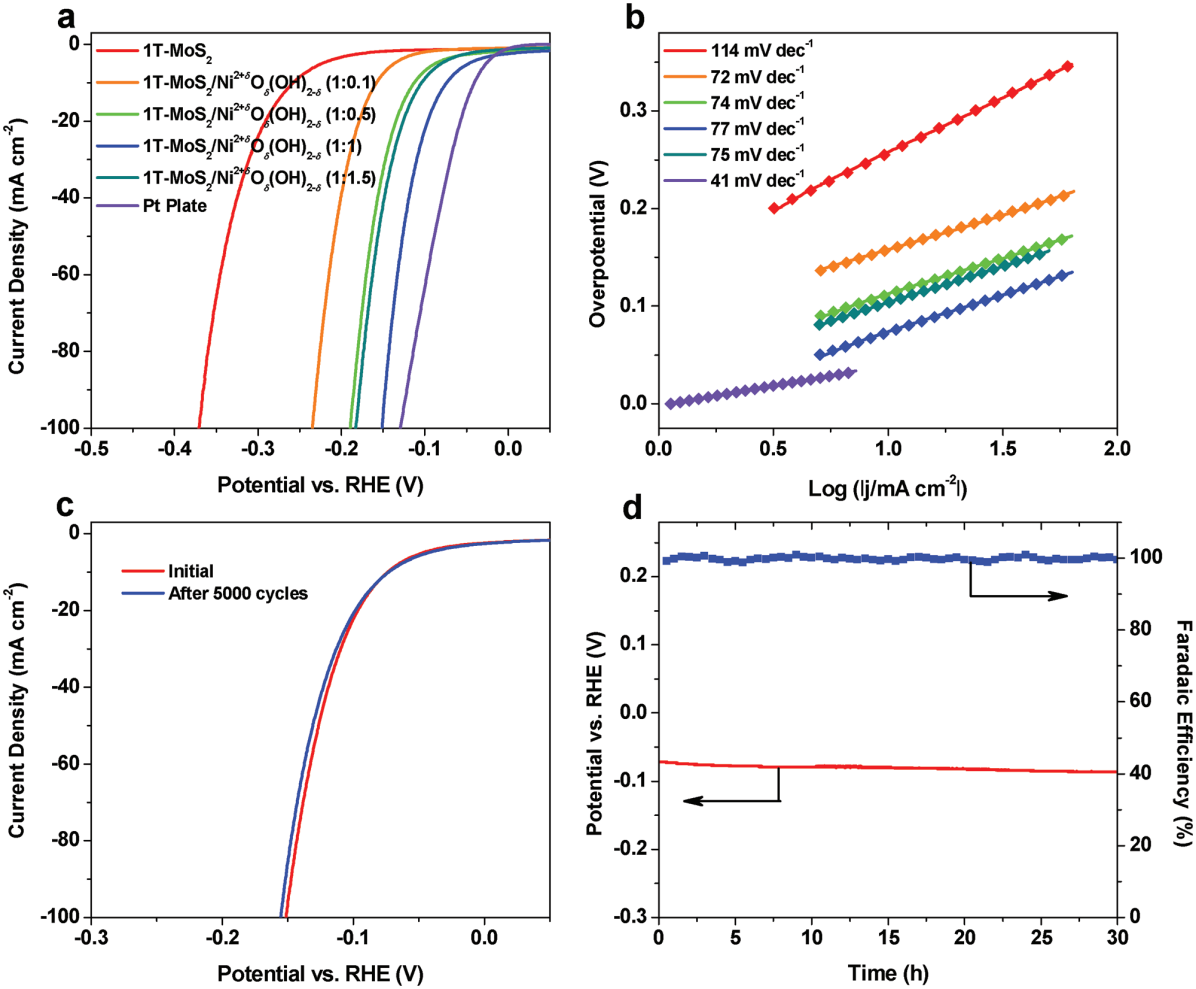
HER performance of the 1T‐MoS_2_/Ni^2+^ *^δ^*O*_δ_*(OH)_2−_ *_δ_* hybrids in 1 m KOH. a) Polarization curves. b) Tafel plots. c) Polarization curves for evaluating the accelerated durability of 1T‐MoS_2_/Ni^2+^ *^δ^*O*_δ_*(OH)_2−_ *_δ_* (1:1). d) Chronopotentiometric curve recorded at *j* = −10 mA cm^−2^ and H_2_ Faradaic efficiency of 1T‐MoS_2_/Ni^2+^ *^δ^*O*_δ_*(OH)_2−_ *_δ_* (1:1) recorded simultaneously.

The HER performance of the 1T‐MoS_2_ and 1T‐MoS_2_/Ni^2+^ *^δ^*O*_δ_*(OH)_2−_ *_δ_* hybrids was first evaluated in 1 m KOH aqueous electrolyte. All the catalyst electrodes were prepared by drop‐drying the catalyst inks onto carbon fiber paper (CFP) with a mass loading of 0.4 mg cm^−2^ based on the mass of MoS_2_ (see details in the Experimental Section). CFP is chosen as the conductive substrate because it is porous and has negligible HER activity in the surveyed potential region in this work (Figure S8, Supporting Information). **Figure** **2**a shows the polarization curves of these catalyst electrodes along with a benchmark Pt plate electrode at a scan rate of 5 mV s^−1^. Compared with 1T‐MoS_2_, the 1T‐MoS_2_/Ni^2+^ *^δ^*O*_δ_*(OH)_2−_ *_δ_* hybrids exhibit large positive shift of the polarization curves, suggesting significant enhancement of the catalytic activities. The mass ratio of 1T‐MoS_2_ to Ni^2+^ *^δ^*O*_δ_*(OH)_2−_ *_δ_* has substantial influence on the catalytic activities of the hybrid catalysts (Figure 2a). The best performance is achieved in the 1T‐MoS_2_/Ni^2+^ *^δ^*O*_δ_*(OH)_2−_ *_δ_* (1:1) catalyst electrode where an overpotential of ≈73 mV is needed to drive a cathodic current density of 10 mA cm^−2^, about 185 mV less than that of 1T‐MoS_2_ and just ≈32 mV more than that of benchmark Pt plate. Such activity surpasses most molybdenum‐based or metal sulfides HER electrocatalysts reported so far and is among the best nonprecious metal‐based electrocatalysts in alkaline media (Table S1, Supporting Information). Especially, when 1T‐MoS_2_/Ni^2+^ *^δ^*O*_δ_*(OH)_2−_ *_δ_* (1:1) catalyst was deposited on porous nickel foam substrate with a higher mass loading of ≈4 mg cm^−2^, an overpotential of ≈43 and ≈103 mV is needed to drive a current density of 10 and 100 mA cm^−2^, respectively (Figure S9, Supporting Information). Electrochemical impedance spectra of the 1T‐MoS_2_ and the hybrid catalyst electrodes indicate that the improved HER activities in the hybrids are due to the faster electrode reaction kinetics (Figure S10, Supporting Information). The Tafel slope of 1T‐MoS_2_ is determined to be ≈114 mV dec^−1^, suggesting that the rate‐limiting step is the Volmer reaction of HER in alkaline media (Figure 2b).^38^ However, the Tafel slopes of all the hybrid catalyst electrodes are around 75 mV dec^−1^, much smaller than that of 1T‐MoS_2_, suggesting significantly improved reaction kinetics of HER in alkaline media.

The durability of the 1T‐MoS_2_/Ni^2+^ *^δ^*O*_δ_*(OH)_2−_ *_δ_* (1:1) catalyst electrode was further evaluated by accelerated degradation test and chronopotentiometry. After 5000 continuous cyclic voltammograms between −0.2 and 0.05 V versus reversible hydrogen electrode (RHE) at an accelerated scan rate of 100 mV s^−1^, the hybrid catalyst electrode shows excellent HER activity with negligible degradation (Figure 2c). The chronopotentiometric curve recorded at a constant cathodic current density of 10 mA cm^−2^ over a course of 30 h shows a slight increase of the overpotential of only 12 mV, also demonstrating the excellent catalytic durability of the hybrid catalyst electrode (Figure 2d). The morphology of the post‐HER sample was further checked by TEM and EDS, which reveal the maintenance of the 2D nanosheet structure and uniform distribution of Ni^2+^ *^δ^*O*_δ_*(OH)_2−_ *_δ_* over the MoS_2_ nanosheets (Figure S11, Supporting Information). XRD and XPS results of the post‐HER electrode demonstrate no noticeable changes in structure and the chemical states of the hybrid compared to the as‐prepared hybrid (Figure S12, Supporting Information). The H_2_ Faradaic efficiency during the period of chronopotentiometry test was determined to be ≈100% by a coupled gas chromatograph monitored every 30 min (Figure 2d). Besides, there is no degradation of the catalytic performance for the hybrid catalyst after being stored in ethanol for over three months (Figure S13, Supporting Information). These above results indicate the excellent stabilities of the 1T‐MoS_2_/Ni^2+^ *^δ^*O*_δ_*(OH)_2−_ *_δ_* hybrid catalysts for HER in alkaline media.

Like the case in alkaline media, significant improvement of HER activity caused by the decoration of Ni^2+^ *^δ^*O*_δ_*(OH)_2−_ *_δ_* on 1T‐MoS_2_ is also observed in neutral media. To drive a cathodic current density of 10 mA cm^−2^ in 1 m phosphate buffer solution (PBS), an overpotential of ≈153 mV is needed for the 1T‐MoS_2_/Ni^2+^ *^δ^*O*_δ_*(OH)_2−_ *_δ_* (1:1) catalyst electrode, about 133 mV less than that for the 1T‐MoS_2_ catalyst electrode (Figure S14, Supporting Information). It can be ranked among the top series of the recently reported neutral HER catalysts (Table S2, Supporting Information).

**Figure 3:**
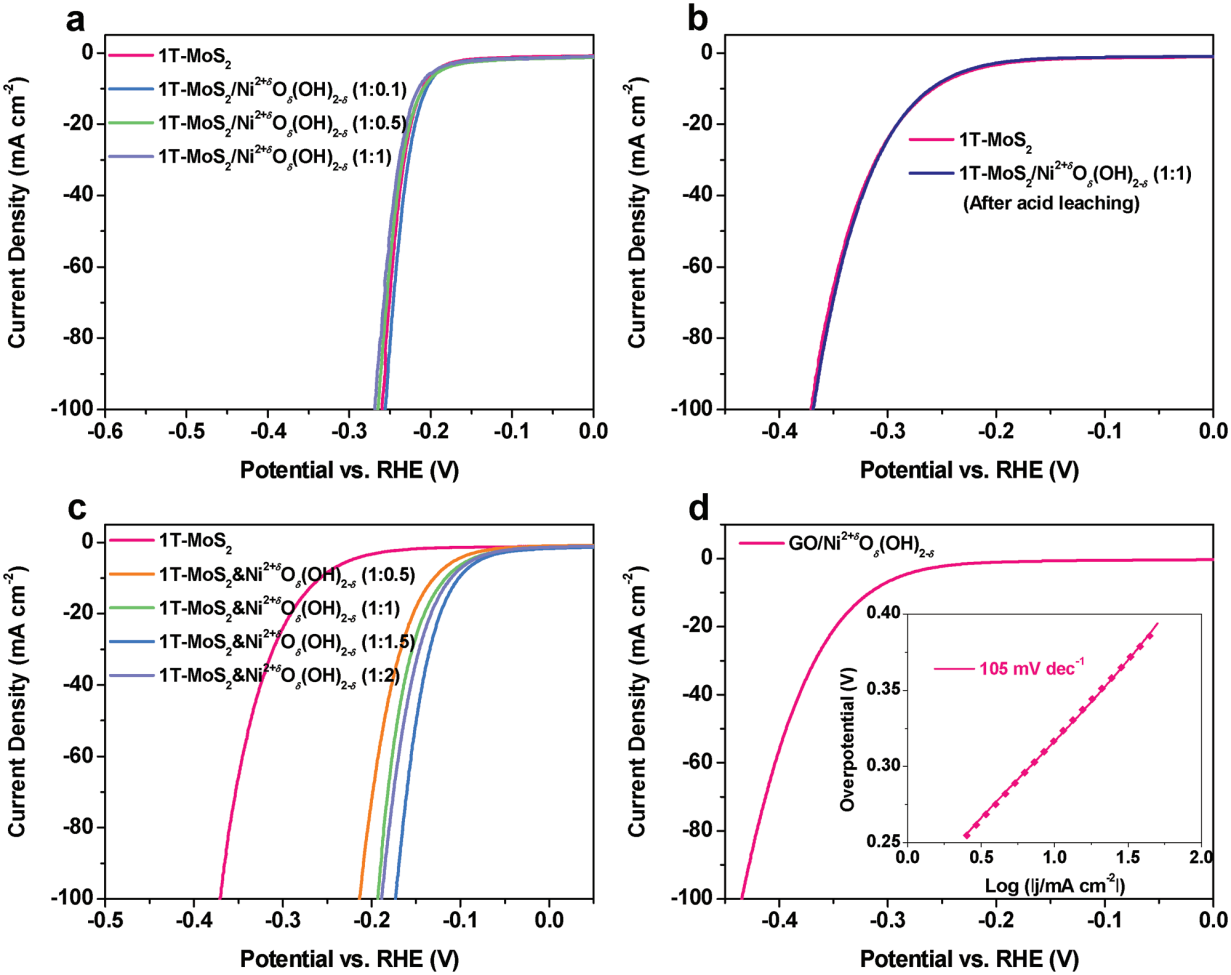
Roles of Ni^2+^ *^δ^*O*_δ_*(OH)_2−_ *_δ_* on the HER activity of the hybrid. a) Polarization curves of the 1T‐MoS_2_/Ni^2+^ *^δ^*O*_δ_*(OH)_2−_ *_δ_* hybrids in acidic 0.5 m H_2_SO_4_. b) Comparison of the HER activities between 1T‐MoS_2_ and the acid leached 1T‐MoS_2_/Ni^2+^ *^δ^*O*_δ_*(OH)_2−_ *_δ_* (1:1) hybrid in 1 m KOH. c) Polarization curves of the physical mixtures of 1T‐MoS_2_ nanosheets and Ni^2+^ *^δ^*O*_δ_*(OH)_2−_ *_δ_* nanoparticles with different mass ratio in 1 m KOH. d) Polarization curve and Tafel plots (inset) of the GO/Ni^2+^ *^δ^*O*_δ_*(OH)_2−_ *_δ_* hybrid with mass ratio of 1:1 in 1 m KOH.

A series of control experiments were further performed to identify the roles that Ni^2+^ *^δ^*O*_δ_*(OH)_2−_ *_δ_* plays on the improved HER performance of the hybrid catalysts. Previous work has demonstrated that doping of transition metal atoms such as Ni or Co into MoS_2_ can significantly enhance the HER activities of the doped MoS_2_ catalysts in both acidic and alkaline media.^31^, ^52^ To check whether the catalytic HER performance of our hybrid catalysts is affected by Ni doping, the electrocatalytic HER activities of the 1T‐MoS_2_/Ni^2+^ *^δ^*O*_δ_*(OH)_2−_ *_δ_* hybrids were further evaluated in acidic electrolytes. It can be found that all the 1T‐MoS_2_/Ni^2+^ *^δ^*O*_δ_*(OH)_2−_ *_δ_* hybrids show similar HER activities compared to 1T‐MoS_2_ in acidic 0.5 m H_2_SO_4_ electrolyte (**Figure** **3**a). The Tafel slopes of all the hybrid catalyst electrodes are determined to be around 50 mV dec^−1^, similar to the one of 1T‐MoS_2_ (Figure S15, Supporting Information). These results indicate that there is no noticeable effect of Ni doping on the HER activities of the hybrid catalysts in acidic solution. Further, an acid leaching process was conducted on the 1T‐MoS_2_/Ni^2+^ *^δ^*O*_δ_*(OH)_2−_ *_δ_* (1:1) hybrid and nickel‐containing species could be completely removed in the process as evidenced by EDS (Figure S16, Supporting Information). Inductively coupled plasma‐mass spectroscopy (ICP‐MS) result shows negligible amount of nickel detected, also suggesting the complete dissolution of Ni^2+^ *^δ^*O*_δ_*(OH)_2−_ *_δ_* by acid leaching. The HER performance of the acid leached catalyst is almost same as 1T‐MoS_2_ in 1 m KOH electrolyte (Figure 3b), suggesting that Ni^2+^ *^δ^*O*_δ_*(OH)_2−_ *_δ_* is the key species in improving the HER performance of the hybrid in alkaline media. All the above results could exclude the possibility that nickel doping in 1T‐MoS_2_ lattice contributes to the excellent HER performance of the 1T‐MoS_2_/Ni^2+^ *^δ^*O*_δ_*(OH)_2−_ *_δ_* hybrids.

The physical mixtures of 1T‐MoS_2_ nanosheets and Ni^2+^ *^δ^*O*_δ_*(OH)_2−_ *_δ_* nanoparticles (defined as 1T‐MoS_2_&Ni^2+^ *^δ^*O*_δ_*(OH)_2−_ *_δ_*) also show notably improved HER activities compared to 1T‐MoS_2_ (Figure 3c). The Tafel slopes of these physical mixtures are also around 75 mV dec^−1^, similar to those of the 1T‐MoS_2_/Ni^2+^ *^δ^*O*_δ_*(OH)_2−_ *_δ_* hybrids (Figure S17, Supporting Information). This is also a strong evidence that the improved HER activities and kinetics of the 1T‐MoS_2_/Ni^2+^ *^δ^*O*_δ_*(OH)_2−_ *_δ_* hybrids are mainly due to the effect caused by the decoration of Ni^2+^ *^δ^*O*_δ_*(OH)_2−_ *_δ_*, instead of doping or other nickel sulfide species formed on 1T‐MoS_2_. Among these physical mixtures, the best performance is achieved in 1T‐MoS_2_&Ni^2+^ *^δ^*O*_δ_*(OH)_2−_ *_δ_* (1:1.5). However, to achieve an HER current density of 10 mA cm^−2^, the 1T‐MoS_2_&Ni^2+^ *^δ^*O*_δ_*(OH)_2−_ *_δ_* (1:1.5) and 1T‐MoS_2_&Ni^2+^ *^δ^*O*_δ_*(OH)_2−_ *_δ_* (1:1) require an overpotential of 102 and 116 mV, respectively. These are significantly larger than that of the 1T‐MoS_2_/Ni^2+^ *^δ^*O*_δ_*(OH)_2−_ *_δ_* (1:1) hybrid (73 mV), suggesting the superior performance of the hybrid.

To identify whether Ni^2+^ *^δ^*O*_δ_*(OH)_2−_ *_δ_* could act as the active species for the excellent HER performance of the 1T‐MoS_2_/Ni^2+^ *^δ^*O*_δ_*(OH)_2−_ *_δ_* hybrids, a graphene oxide (GO)/Ni^2+^ *^δ^*O*_δ_*(OH)_2−_ *_δ_* hybrid composed of mildly oxidized GO nanosheets and Ni^2+^ *^δ^*O*_δ_*(OH)_2−_ *_δ_* with mass ratio of 1:1 was prepared by the same method for the synthesis of 1T‐MoS_2_/Ni^2+^ *^δ^*O*_δ_*(OH)_2−_ *_δ_* (1:1) hybrid (Figure S18, Supporting Information). This GO/Ni^2+^ *^δ^*O*_δ_*(OH)_2−_ *_δ_* (1:1) hybrid catalyst electrode shows low HER activity, with an overpotential of ≈316 mV for achieving a cathodic current density of 10 mA cm^−2^ (Figure 3d). The Tafel slope of 105 mV dec^−1^ is also larger than that of the 1T‐MoS_2_/Ni^2+^ *^δ^*O*_δ_*(OH)_2−_ *_δ_* (1:1) hybrid. Considering that both 1T‐MoS_2_ and mildly oxidized GO have good electrical conductivity and large surface area, the huge difference of HER performance between 1T‐MoS_2_/Ni^2+^ *^δ^*O*_δ_*(OH)_2−_ *_δ_* (1:1) and GO/Ni^2+^ *^δ^*O*_δ_*(OH)_2−_ *_δ_* (1:1) can reasonably exclude the possibility that Ni^2+^ *^δ^*O*_δ_*(OH)_2−_ *_δ_* is the active species for HER in 1T‐MoS_2_/Ni^2+^ *^δ^*O*_δ_*(OH)_2−_ *_δ_* in alkaline media, consistent with the results in previous work.^62^, ^63^ Therefore, all the results from the above control experiments indicate that Ni^2+^ *^δ^*O*_δ_*(OH)_2−_ *_δ_* in the 1T‐MoS_2_/Ni^2+^ *^δ^*O*_δ_*(OH)_2−_ *_δ_* hybrids acts as an cocatalyst with 1T‐MoS_2_ to facilitate HER in alkaline and neutral electrolytes.

In addition, the effect of Ni^2+^ *^δ^*O*_δ_*(OH)_2−_ *_δ_* decoration on the HER activity of semiconducting 2H‐MoS_2_ was also investigated. The 2H‐MoS_2_ was prepared by a hydrothermal method according to a reported literature,^55^ and it exhibits similar morphology as 1T‐MoS_2_ but characteristic 2H phase (Figure S19, Supporting Information).^44^, ^55^ 2H‐MoS_2_/Ni^2+^ *^δ^*O*_δ_*(OH)_2−_ *_δ_* (1:1) hybrid was synthesized by just replacing 1T‐MoS_2_ with 2H‐MoS_2_ in the hybrid preparation. TEM and EDS elemental mapping characterizations on the 2H‐MoS_2_/Ni^2+^ *^δ^*O*_δ_*(OH)_2−_ *_δ_* (1:1) hybrid demonstrate the uniform hybridization between 2H‐MoS_2_ and Ni^2+^ *^δ^*O*_δ_*(OH)_2−_ *_δ_* (Figure S20, Supporting Information). Electrochemical characterizations in 1 m KOH electrolyte show that the Ni^2+^ *^δ^*O*_δ_*(OH)_2−_ *_δ_* decoration can also significantly improve the HER activities and reaction kinetic in view of the substantially positive shift of the polarization curve and smaller Tafel slope of the 2H‐MoS_2_/Ni^2+^ *^δ^*O*_δ_*(OH)_2−_ *_δ_* (1:1) hybrid compared to those of the 2H‐MoS_2_ (Figure S21, Supporting Information). However, the HER activity of the 2H‐MoS_2_/Ni^2+^ *^δ^*O*_δ_*(OH)_2−_ *_δ_* (1:1) hybrid is worse than the 1T‐MoS_2_/Ni^2+^ *^δ^*O*_δ_*(OH)_2−_ *_δ_* (1:1) hybrid. Two differences between 2H‐MoS_2_ and 1T‐MoS_2_ can be regarded as the main reasons for this result: (1) 1T‐MoS_2_ owns HER active sites at both the edges and basal planes while only the metallic edge sites are active for HER in 2H‐MoS_2_; (2) 1T‐MoS_2_ has much better electrical conductivity than 2H‐MoS_2_.^7^, ^45^, ^48^

**Figure 4:**
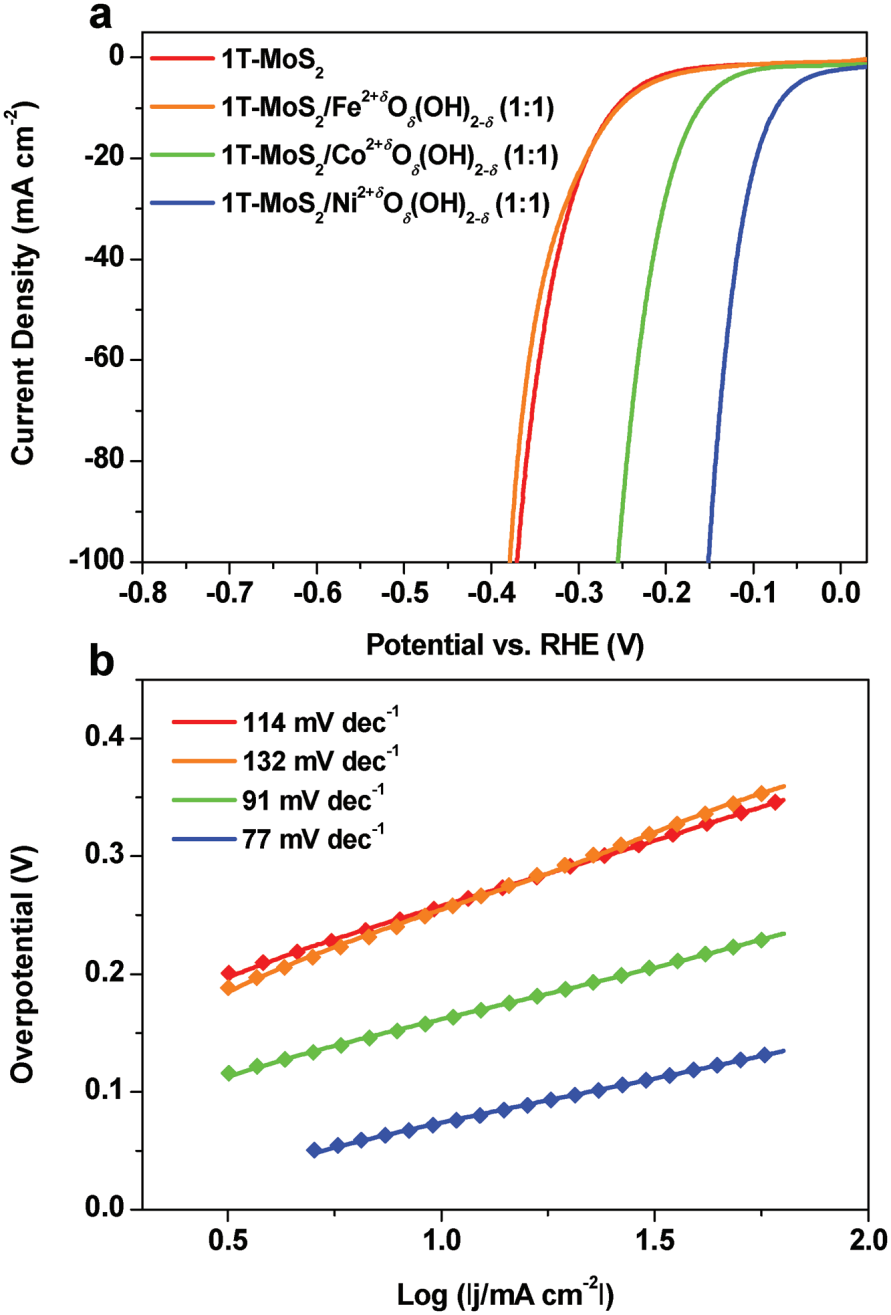
Effect of different metal hydr(oxy)oxides on the HER activity. a) Polarization curves and b) Tafel plots of the 1T‐MoS_2_/M^2+^ *^δ^*O*_δ_*(OH)_2−_ *_δ_* (1:1) hybrid catalyst electrodes (M = Fe, Co, Ni).

The effects of different 3d‐M hydr(oxy)oxides (3d‐M = Fe, Co, and Ni) on the HER activity of the hybrid were also studied. The synthesis of the 1T‐MoS_2_/Fe^2+^ *^δ^*O*_δ_*(OH)_2−_ *_δ_* (1:1) and 1T‐MoS_2_/Co^2+^ *^δ^*O*_δ_*(OH)_2−_ *_δ_* (1:1) hybrids was similar to that of 1T‐MoS_2_/Ni^2+^ *^δ^*O*_δ_*(OH)_2−_ *_δ_* (1:1) by just replacing NiCl_2_ with FeCl_3_ or CoCl_2_, respectively. TEM and EDS elemental mapping results reveal the similar nanosheet structure of the hybrids and uniform hybridization of Fe^2+^ *^δ^*O*_δ_*(OH)_2−_ *_δ_* or Co^2+^ *^δ^*O*_δ_*(OH)_2−_ *_δ_* with 1T‐MoS_2_ (Figures S22 and S23, Supporting Information). Electrochemical characterization results of these hybrids for HER in 1 m KOH electrolyte are summarized in **Figure** **4**. It can be found that the 1T‐MoS_2_/Fe^2+^ *^δ^*O*_δ_*(OH)_2−_ *_δ_* (1:1) hybrid shows similar HER activity as 1T‐ MoS_2_. However, an obvious activity enhancement is achieved on the 1T‐MoS_2_/Co^2+^ *^δ^*O*_δ_*(OH)_2−_ *_δ_* (1:1) hybrid, but not as good as the 1T‐MoS_2_/Ni^2+^ *^δ^*O*_δ_*(OH)_2−_ *_δ_* (1:1) hybrid (Figure 4a). The Tafel slopes of these catalyst electrodes follow the order: 1T‐MoS_2_/Fe^2+^ *^δ^*O*_δ_*(OH)_2−_ *_δ_* (1:1) > 1T‐MoS_2_ > 1T‐MoS_2_/Co^2+^ *^δ^*O*_δ_*(OH)_2−_ *_δ_* (1:1) > 1T‐MoS_2_/Ni^2+^ *^δ^*O*_δ_*(OH)_2−_ *_δ_* (1:1) (Figure 4b). It has been indicated that the OH‐M^2+^ *^δ^* bond strength is an activity descriptor to evaluate the reactivity for adsorption and dissociation of water on the surface of 3d‐M hydr(oxy)oxides.^9^ The trend of the HER activity of these hybrid catalysts inversely tracks the OH‐M^2+^ *^δ^* bond strength order that OH‐Fe^2+^ *^δ^* > OH‐Co^2+^ *^δ^* > OH‐Ni^2+^ *^δ^*, suggesting that the overall HER rate is controlled by optimizing the water adsorption and dissociation elementary reaction.^9^ According to the standard Brønsted–Evans–Polanyi‐type principles, the binding strength of OH‐M^2+^ *^δ^* neither too strong nor too weak will give a favorable balance between facilitating water dissociation and suppressing “poisoning” by OH_ad_ (product from water dissociation) on M^2+^ *^δ^*O*_δ_*(OH)_2−_ *_δ_*.^4^, ^9^, ^64^ As demonstrated here, the best combination among these 3d‐M hydr(oxy)oxides with 1T‐MoS_2_ is 1T‐MoS_2_/Ni^2+^ *^δ^*O*_δ_*(OH)_2−_ *_δ_* (1:1), suggesting that Ni^2+^ *^δ^*O*_δ_*(OH)_2−_ *_δ_* owns the optimum binding energies to H_2_O and OH_ad_ and thus the highest activity for water dissociation. Although Co^2+^ *^δ^*O*_δ_*(OH)_2−_ *_δ_* and Fe^2+^ *^δ^*O*_δ_*(OH)_2−_ *_δ_* own stronger binding strength to H_2_O than Ni^2+^ *^δ^*O*_δ_*(OH)_2−_ *_δ_*, the stronger adsorption of OH_ad_ on Co and Fe hydr(oxy)oxides could block the readsorption of H_2_O at the same adsorption sites, resulting in lower activities for the water dissociation process.

**Figure 5:**
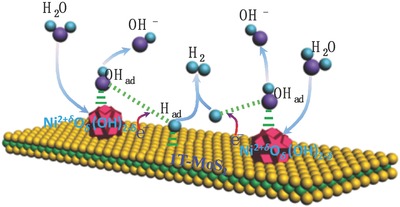
HER catalysis mechanism on the 1T‐MoS_2_/Ni^2+^ *^δ^*O*_δ_*(OH)_2−_ *_δ_* hybrid. Schematic showing HER process occurred on the 1T‐MoS_2_/Ni^2+^ *^δ^*O*_δ_*(OH)_2−_ *_δ_* hybrid through a bifunctional mechanism in alkaline or neutral media.

All the above results and discussions reveal that excellent HER performance of the binary 1T‐MoS_2_/Ni^2+^ *^δ^*O*_δ_*(OH)_2−_ *_δ_* hybrid is attributed to the adopted bifunctional mechanism taking place at the three‐phase boundaries among the electrolyte, Ni^2+^ *^δ^*O*_δ_*(OH)_2−_ *_δ_* and 1T‐MoS_2_ (**Figure** **5**). Both 1T‐MoS_2_ and Ni^2+^ *^δ^*O*_δ_*(OH)_2−_ *_δ_* play specific roles in different elementary reactions with their own advantages to synergistically improve the overall HER kinetic. Ni^2+^ *^δ^*O*_δ_*(OH)_2−_ *_δ_* nanoparticles with optimum binding strength to H_2_O and OH_ad_ facilitate the adsorption and dissociation of H_2_O to supply protons to the nearby active sites on 1T‐MoS_2_, while 1T‐MoS_2_ nanosheets contribute to the electron transfer, adsorption, and combination of H intermediates and desorption of H_2_ through a Heyrovsky step. After being aware of the HER mechanism, we can understand the effect of mass ratio of 1T‐MoS_2_ to Ni^2+^ *^δ^*O*_δ_*(OH)_2−_ *_δ_* on the HER activities of the hybrids. The electrochemical accessible surface area (ECSA) of the 1T‐MoS_2_/Ni^2+^ *^δ^*O*_δ_*(OH)_2−_ *_δ_* hybrid electrodes is analyzed from the electrochemical double‐layer capacitances of the hybrid catalyst electrodes in the non‐Faradaic potential region (0.1–0.2 V vs RHE). The free growing Ni^2+^ *^δ^*O*_δ_*(OH)_2−_ *_δ_* exhibits much smaller double‐layer capacitance than 1T‐MoS_2_ (Figure S24, Supporting Information). So the decrease of ECSA with higher content of Ni^2+^ *^δ^*O*_δ_*(OH)_2−_ *_δ_* in the hybrids is ascribed to the coverage of Ni^2+^ *^δ^*O*_δ_*(OH)_2−_ *_δ_* on 1T‐MoS_2_ (Figures S24 and S25, Supporting Information). It should be noted that the catalytic activity for HER is related to the active sites at the electrochemical accessible boundaries between 1T‐MoS_2_ and Ni^2+^ *^δ^*O*_δ_*(OH)_2−_ *_δ_*. Such accessible boundaries increase with the Ni^2+^ *^δ^*O*_δ_*(OH)_2−_ *_δ_* content in the hybrid when the mass ratio of Ni^2+^ *^δ^*O*_δ_*(OH)_2−_ *_δ_* to 1T‐MoS_2_ is low, despite the decrease of the surface area of 1T‐MoS_2_. However, when the mass ratio surpasses an optimum value (1:1 in mass for the hybrid), the catalytically active boundaries will decrease with the Ni^2+^ *^δ^*O*_δ_*(OH)_2−_ *_δ_* increase because the accessible surface area of 1T‐MoS_2_ becomes a limiting factor (as illustrated in Figures S26 and S27, Supporting Information). Therefore, the influence of Ni^2+^ *^δ^*O*_δ_*(OH)_2−_ *_δ_* content on the catalytically accessible boundaries of the hybrid catalyst electrode can well explain the HER activities trend observed in the 1T‐MoS_2_/Ni^2+^ *^δ^*O*_δ_*(OH)_2−_ *_δ_* hybrids with various ratio. The superior HER performance of the in situ formed hybrids over the physical mixtures is accordingly due to the more uniform decoration of the Ni^2+^ *^δ^*O*_δ_*(OH)_2−_ *_δ_* nanoparticles on the surface of 1T‐MoS_2_ in the in situ synthesis process than simple physical mixing.

To quantify the turnover frequency toward HER of the 1T‐MoS_2_/3d‐M^2+^ *^δ^*O*_δ_*(OH)_2−_ *_δ_* (1:1) hybrid catalysts is meaningful to clearly compare their intrinsic catalytic activities for HER. However, there is no suitable method to determine the number of electrochemically accessible HER active sites of these hybrid catalysts currently because the HER is related to the active sites at the electrochemical accessible boundaries between 1T‐MoS_2_ and Ni^2+^ *^δ^*O*_δ_*(OH)_2−_ *_δ_*. Also, 3d‐M^2+^ *^δ^*O*_δ_*(OH)_2−_ *_δ_* exhibits significantly different capacity behavior in the double‐layer capacitance region, resulting in difficult correlation between the ECSA and catalytically accessible boundaries (Figure S28, Supporting Information). Considering the similar particle size of these 3d‐M^2+^ *^δ^*O*_δ_*(OH)_2−_ *_δ_* and their uniform distribution on 1T‐MoS_2_, the significantly different HER activities of the 1T‐MoS_2_/3d‐M^2+^ *^δ^*O*_δ_*(OH)_2−_ *_δ_* (1:1) hybrids could be mainly attributed to the activity difference toward water dissociation of these 3d‐M^2+^ *^δ^*O*_δ_*(OH)_2−_ *_δ_*.

## 3 Conclusion

In summary, we have developed an interface engineering approach to enhance the catalytic activities of MoS_2_ for hydrogen evolution reaction in alkaline or neutral electrolytes by hybridization with Ni^2+^ *^δ^*O*_δ_*(OH)_2−_ *_δ_* nanoparticles. The 1T‐MoS_2_/Ni^2+^ *^δ^*O*_δ_*(OH)_2−_ *_δ_* hybrid is constructed by in situ growing Ni^2+^ *^δ^*O*_δ_*(OH)_2−_ *_δ_* nanoparticles on 1T‐MoS_2_ nanosheets. By tuning the mass ratio of Ni^2+^ *^δ^*O*_δ_*(OH)_2−_ *_δ_* to MoS_2_, the optimized hybrid catalyst can drive a cathodic current density of 10 mA cm^−2^ at an overpotential of 185 mV less than the original 1T‐MoS_2_ in 1 m KOH. The activities of these hybrids outperform most MoS_2_‐based electrocatalysts reported so far. Instead of nickel doping into the MoS_2_ lattice, our results clearly reveal that a bifunctional HER mechanism contributes to the excellent HER activities of the hybrid catalysts. This work highlights the potential of rational design of hybrid structures with distinct functionalities to improve the performance of electrocatalysts. Such strategy can be extended to other systems to develop cost‐effective electrocatalysts with enhanced performance for large‐scale applications.

## 4 Experimental Section

*Synthesis of MoS_2_*: The synthetic process of 1T‐MoS_2_ was referred as a reported literature with slight modification.^47^ In brief, MoO_3_ (36 mg) and urea (360 mg) were first added to water (30 mL) with stirring. Then, 0.5 m thioacetamide aqueous solution (1.2 mL) was added and the mixing solution was stirred for another 2 h. After the stirring, the solution was transferred to a 50 mL autoclave for hydrothermal reaction at 200 °C for 12 h. The resulting product was collected by centrifugation and repeatedly washed with ultrapure water for three times and ethanol for two times and then was stored in ethanol for later use. 2H‐MoS_2_ was prepared by a hydrothermal method from a reported literature.^55^

*Synthesis of the 1T‐MoS_2_/Ni^2+δ^O_δ_(OH)_2_*_−_ *_δ_ Hybrids*: First, a certain volume of 0.2 m NiCl_2_ ethanol solution and NH_4_HCO_3_ (1 mmol) is added into 1T‐MoS_2_ dispersion solution (0.5 mg mL^−1^ in ethanol, 40 mL). After sonication of 5 min, the suspension was magnetically stirred at 400 rpm for 5 h in ambient atmosphere. The products was collected by centrifugation and repeatedly washed with ultrapure water for two times and ethanol for two times and then stored in ethanol for later use.

*Synthesis of the 1T‐MoS_2_* **/**M*^2+δ^O_δ_(OH)_2_*_−_ *_δ_ (1:1) Hybrids, (M = Fe or Co)*: The synthesis procedures were same to those for preparation of the 1T‐MoS_2_/Ni^2+^ *^δ^*O*_δ_*(OH)_2−_ *_δ_* (1:1) hybrid except for replacing NiCl_2_ ethanol solution with 0.2 m CoCl_2_ ethanol solution or 0.2 m FeCl_3_ ethanol solution.

*Synthesis of the GO/Ni^2+δ^O_δ_(OH)_2_*_−_ *_δ_ (1:1) Hybrid*: The synthesis procedures were same to those for preparation of the 1T‐MoS_2_/Ni^2+^ *^δ^*O*_δ_*(OH)_2−_ *_δ_* (1:1) hybrid except for replacing 1T‐MoS_2_ suspension with mildly oxidized GO suspension (0.5 mg mL^−1^ in ethanol, 40 mL). The mildly oxidized GO was prepared according to a previous literature.^65^

*Materials Characterizations*: TEM, STEM imaging and EDS analyses were carried out on an FEI Tecnai G2 F30 transmission electron microscope operated at an accelerated voltage of 300 kV. TEM samples were prepared by drop‐drying catalysts' ethanol suspensions onto copper grids. Raman spectra were taken from the Horiba LabRAM HR Evolution and Jobin Yvon LabRAM Aramis Raman spectrometers. XRD measurements were performed on a Bruker D8 advance powder X‐ray diffractometer using Cu *K*_α_ radiation. XPS measurements were performed on a Perkin–Elmer Model PHI 5600 XPS system with a resolution of 0.3–0.5 eV from a monochromatic aluminum anode X‐ray source. ICP‐MS was performed on an Agilent Technologies 7700 series instrument. The gas products analysis was performed on a gas chromatography (SRI Model 8610C).

*Electrochemical Measurements*: The catalyst inks were prepared by adding 5 wt% Nafion solution (25 µL) into ethanol suspension (975 µL) of catalysts containing MoS_2_ (4 mg) with the assistance of sonication for ≈1 h. The catalyst electrodes were prepared by dropping catalyst ink (50 µL) onto a carbon fiber paper (AvCarb MGL190 from Fuel Cell Store) to cover an area of 0.5 cm^−2^ (mass loading: 0.4 mg cm^−2^ based on the mass of MoS_2_) and dried at room temperature. All electrochemical characterizations were conducted on a CHI 760D electrochemistry workstation. Unless being specifically indicated, all electrochemical tests were performed in a conventional three‐electrode system. A saturated calomel electrode was used as the reference electrode and a graphite rod was used as the counter electrode. 0.5 m H_2_SO_4_, 1 m PBS, and 1 m KOH aqueous solutions were used as the acidic, neutral, and alkaline electrolytes, respectively. For all of the measurements, the corresponding electrolytes were saturated with H_2_ by continuous purging with high‐purity H_2_ (99.999%) during the entire measurement processes. All polarization curves were recorded at a scan rate of 5 mV s^−1^ unless being specifically indicated. H_2_ Faradaic efficiency was quantified referred to a reported method in previous literature except that the CO_2_ flow was replaced by Ar flow (5 sccm).^66^

## Conflict of Interest

The authors declare no conflict of interest.

## Supporting information

Supplementary
