# Supplementary material for: Nickel Hydr(oxy)oxide Nanoparticles on Metallic MoS2 Nanosheets: A Synergistic Electrocatalyst for Hydrogen Evolution Reaction
Source: Adv Sci (Weinh). 2017 Dec 4;5(2):1700644. doi: 10.1002/advs.201700644 (PMC5826986; doi:10.1002/advs.201700644)
Supplement: Supplementary file 1 — Supplementary [file ADVS-5-1700644-s001.pdf]

Copyright WILEY-VCH Verlag GmbH & Co. KGaA, 69469 Weinheim, Germany, 2017.

ADVANCED SCIENCE
Open Access

Supporting Information

for *Adv. Sci.,* DOI: 10.1002/advs.201700644

Nickel Hydr(oxy)oxide Nanoparticles on Metallic MoS_2 Nanosheets: A Synergistic Electrocatalyst for Hydrogen Evolution Reaction

*Xing Zhang and Yongye Liang**

Copyright WILEY-VCH Verlag GmbH & Co. KGaA, 69469 Weinheim, Germany, 2016.

Supporting Information

Nickel Hydr(oxy)oxide Nanoparticles on Metallic MoS_2 Nanosheets: a Synergistic Electrocatalyst for Hydrogen Evolution Reaction

*Xing Zhang, Yongye Liang**

X. Zhang, Prof. Y. Liang

Department of Materials Science and Engineering, Shenzhen Key Laboratory of Printed Organic Electronics, South University of Science and Technology of China, Shenzhen, 518055, China

E-mail: liangyy@sustc.edu.cn

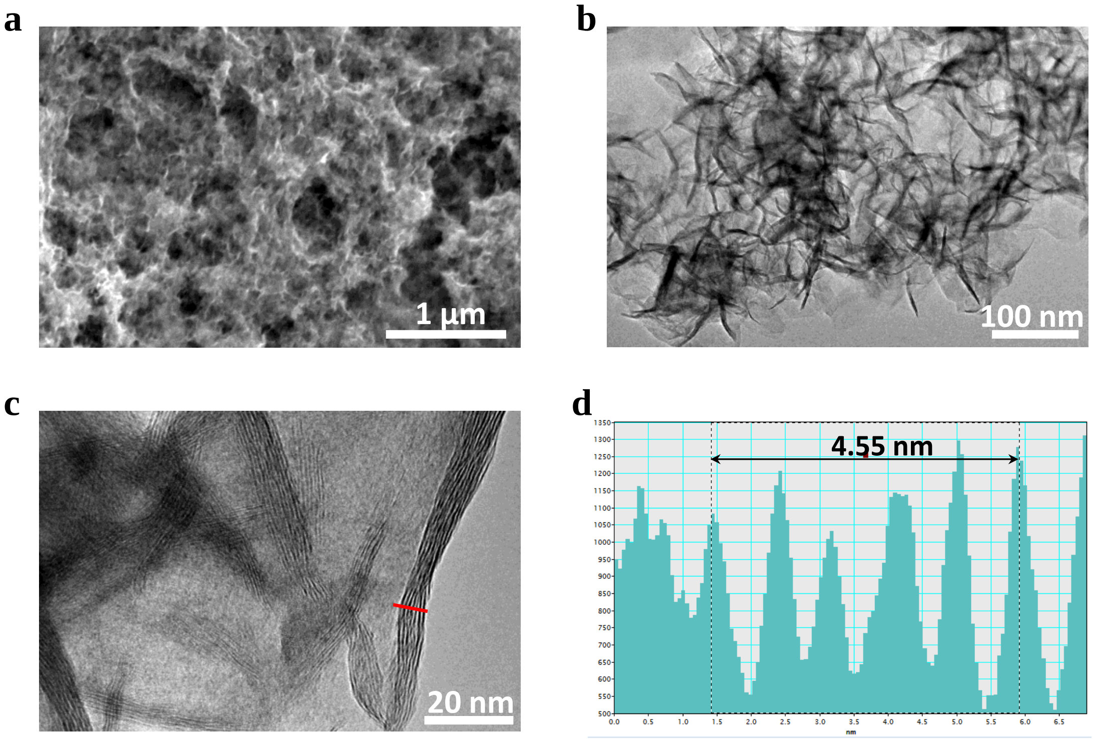

Figure S1. (a) SEM, (b, c) TEM images of the prepared 1T-MoS_2. (d) Line profile along the red line in (c) to show the interlayer spacing of 1T-MoS_2.

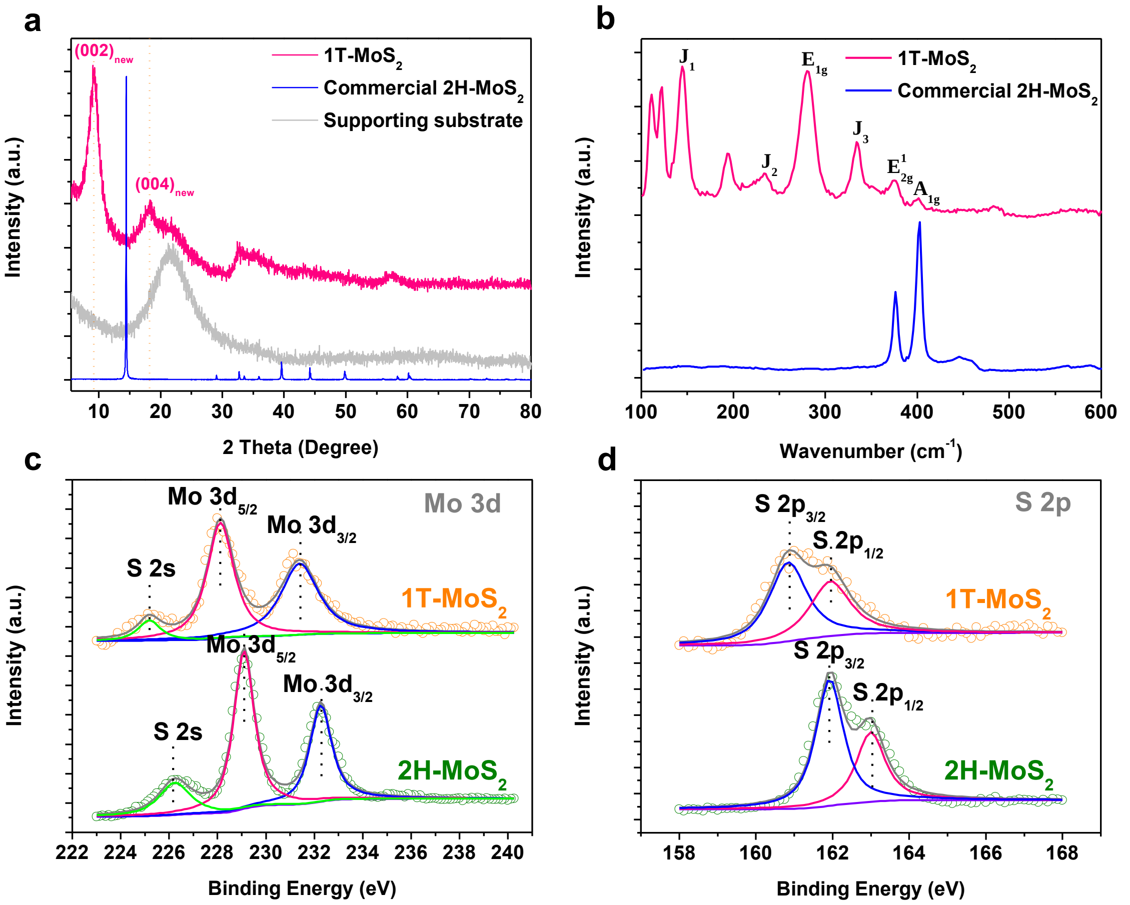

Figure S2. Structure and chemical state analyses of the prepared 1T-MoS_2 with commercial 2H-MoS_2 as the reference. (a) XRD patterns, (b) Raman spectra, (c) Mo 3d and (d) S 2p core level XPS spectra. The background XRD signal from the substrate for supporting the sample is also shown in (a).

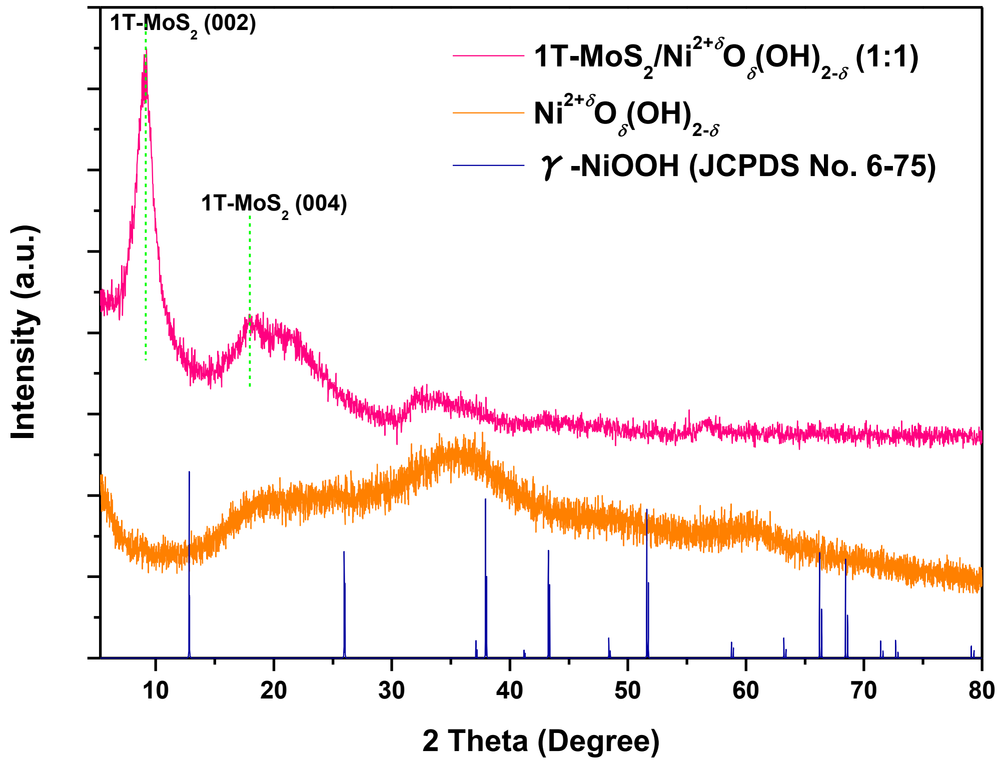

Figure S3. XRD patterns of the 1T-$MoS_2/Ni^{2+\delta}O_\delta(OH)_{2-\delta}$ (1:1) hybrid and the free growing $Ni^{2+\delta}O_\delta(OH)_{2-\delta}$.

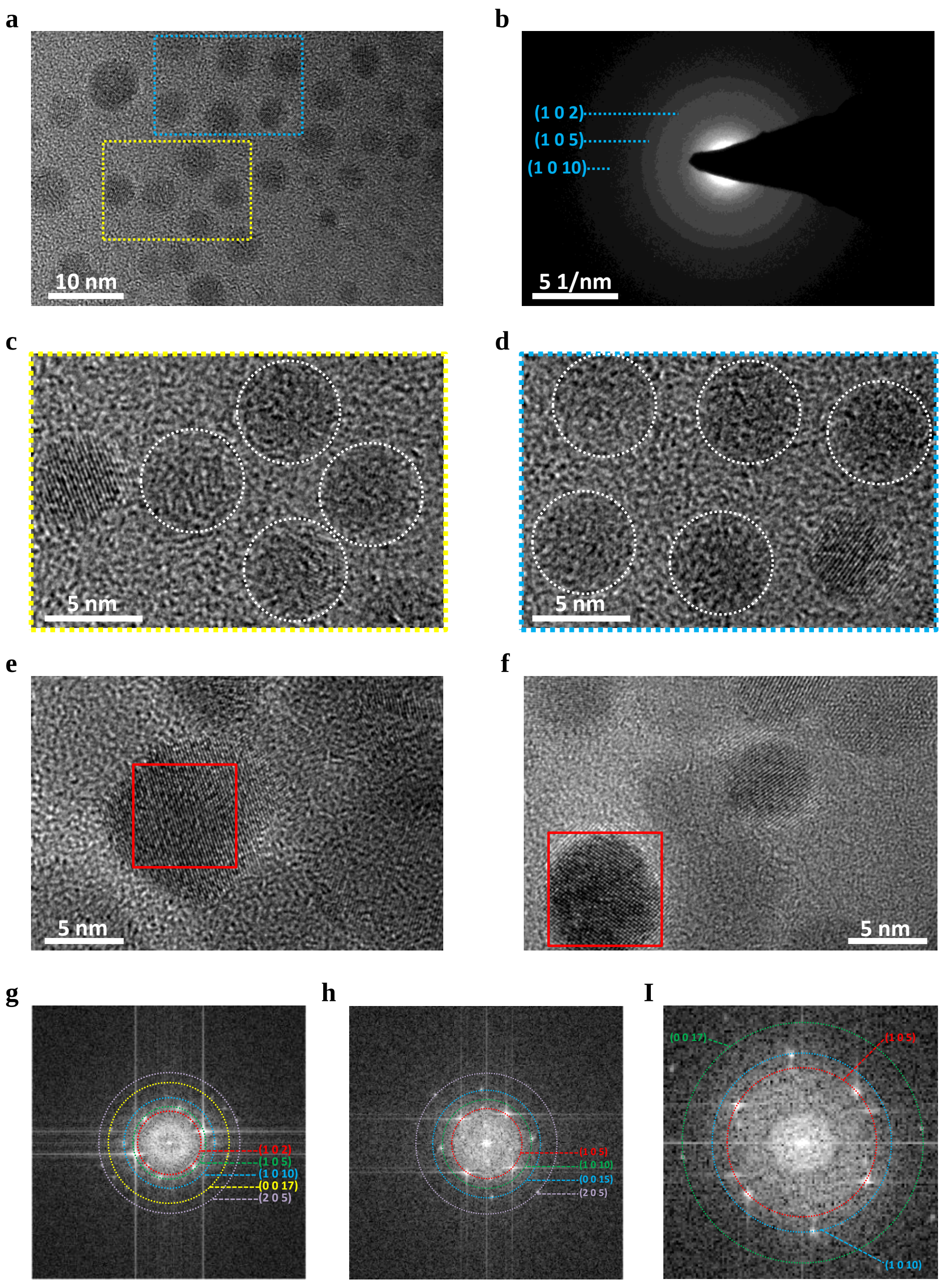

Figure S4. Structure characterization of the free growing $Ni^{2+}{}_{\delta}O_{\delta}(OH)_{2-\delta}$. (a) TEM image. (b) SAED pattern. (c, d) Magnified TEM image of the region marked in (a) to clearly show the crystalline and amorphous nanoparticles (marked with white dotted circle). (e, f) TEM images to show the lattice-resolution nanoparticles. (g, h) FFT pattern of the region marked in (e) and (f), respectively. (I) FFT pattern of one representative crystalline $Ni^{2+}{}_{\delta}O_{\delta}(OH)_{2-\delta}$ nanoparticles in 1T-MoS_2/$Ni^{2+}{}_{\delta}O_{\delta}(OH)_{2-\delta}$ Hybrid. All SAED and FFT patterns are indexed by the standard diffraction pattern of γ-NiOOH (JCPDS No: 6-75).

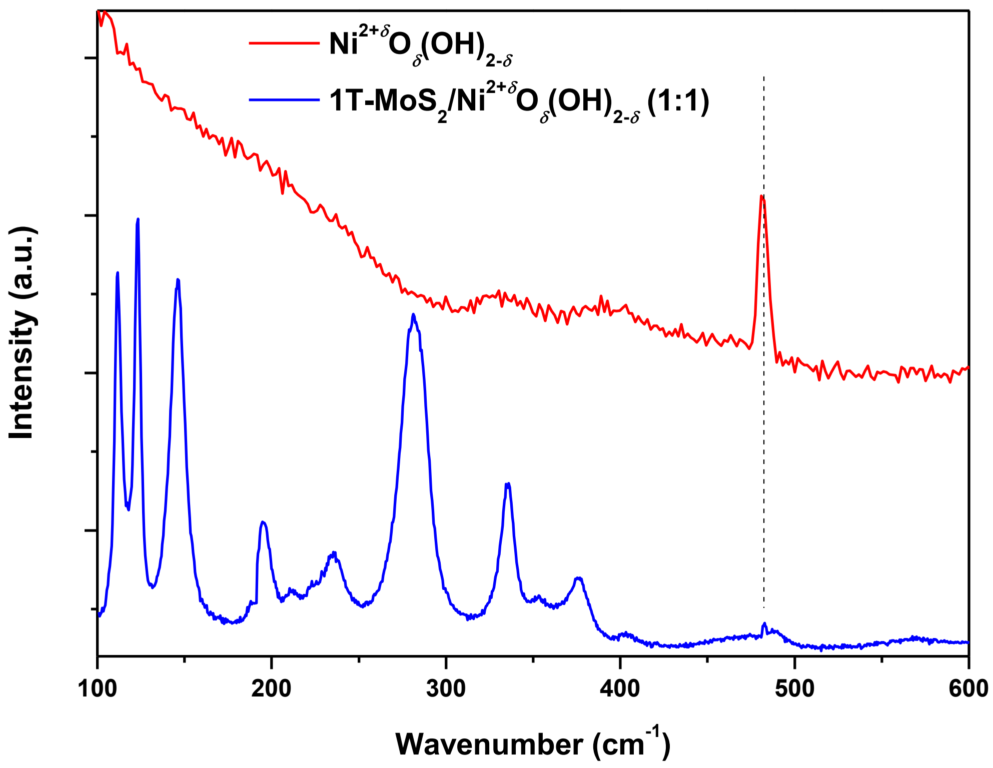

Figure S5. Raman spectra of the $1T-MoS_2/Ni^{2+\delta}O_{\delta(OH)_{2-\delta}}$ (1:1) hybrid and the free growing $Ni^{2+\delta}O_{\delta(OH)_{2-\delta}}$.

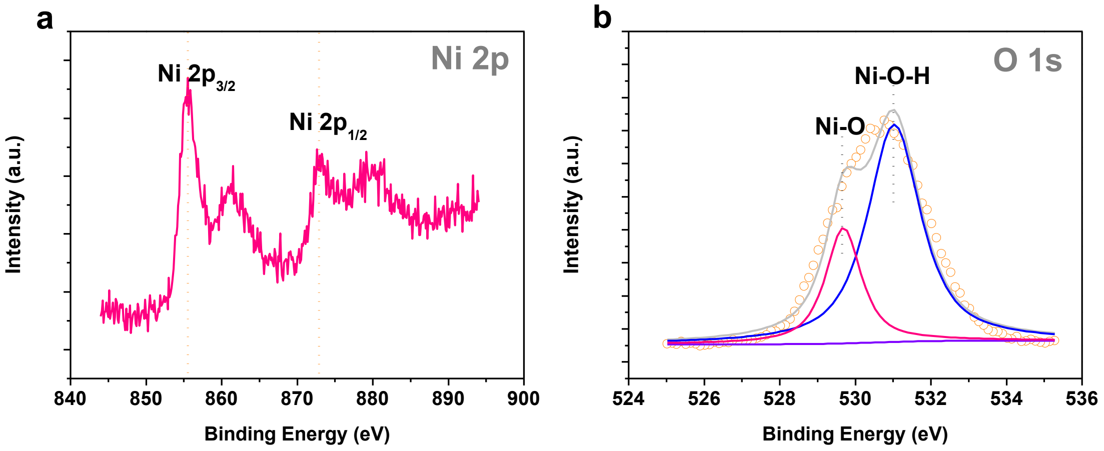

Figure S6. (a) Ni 2p and (b) O 1s core level XPS spectra of the free growing $Ni^{2+}{}_{\delta}O_{\delta}(OH)_{2-\delta}$.

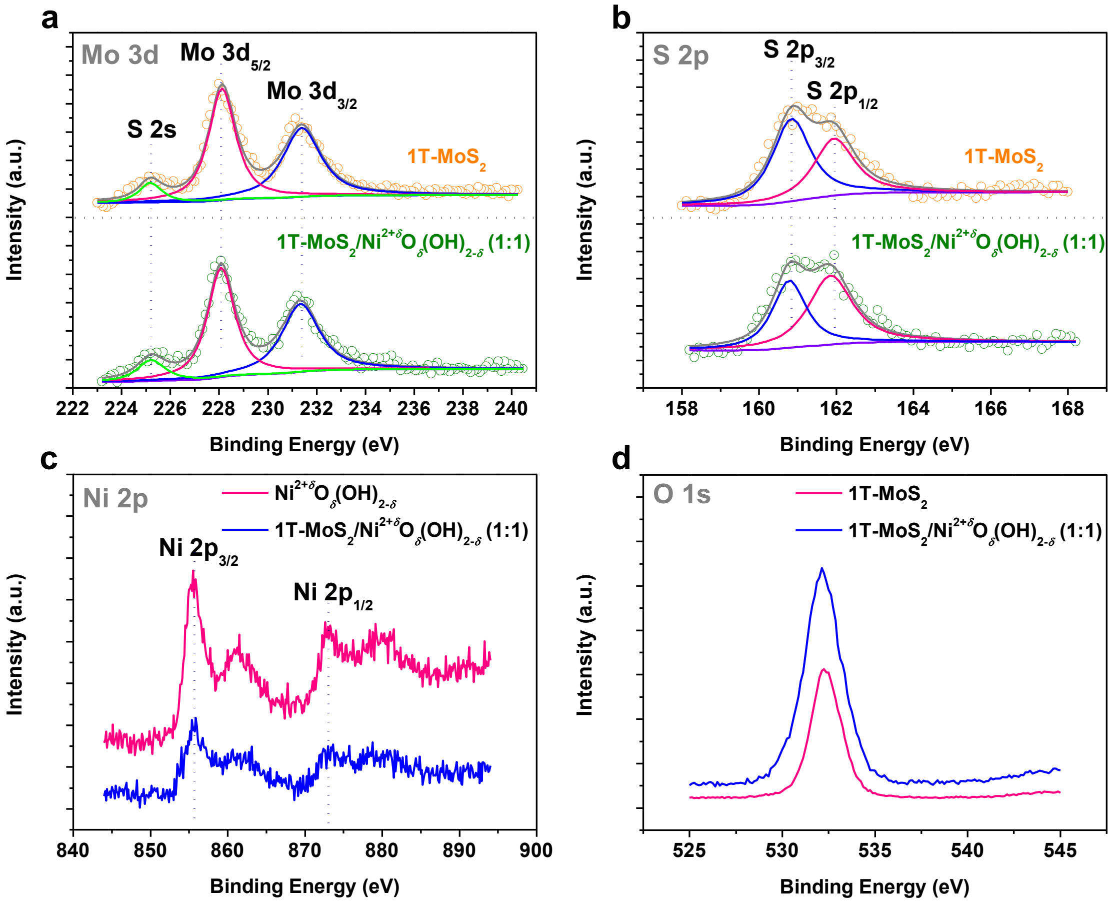

Figure S7. Chemical state analyses on the $1T\text{-}MoS_2/Ni^{2+}{}_{\delta}O_{\delta}(OH)_{2-\delta}$ (1:1) hybrid along with the prepared $1T\text{-}MoS_2$ and the free growing $Ni^{2+}{}_{\delta}O_{\delta}(OH)_{2-\delta}$ as references. (a) Mo 3d, (b) S 2p, (c) Ni 2p, (d) O 1s core level XPS spectra.

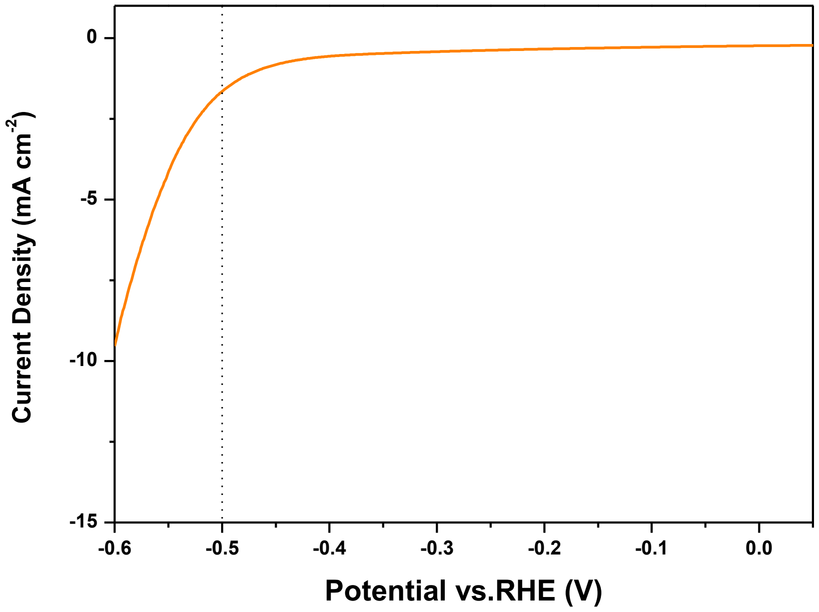

Figure S8. Polarization curve for evaluating the HER activity of bare CFP in 1 M KOH.

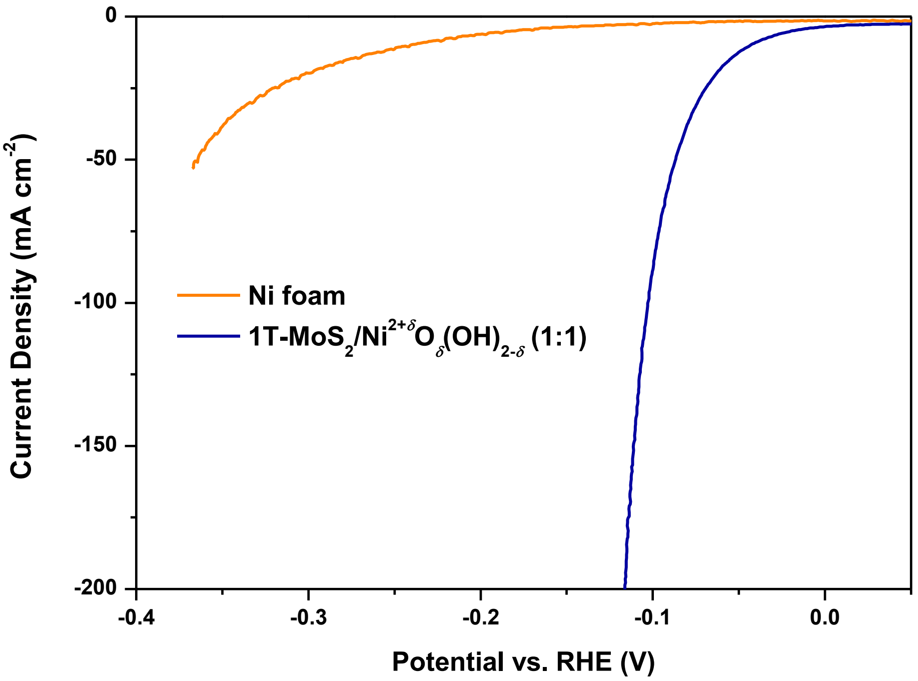

Figure S9. HER performance of 1T-MoS_2/$Ni^{2+\delta}O_{\delta}(OH)_{2-\delta}$ (1:1) on nickel foam substrate with a mass loading of 4 mg cm^{-2} (2 mg cm^{-2} based on 1T-MoS_2) in 1 M KOH.

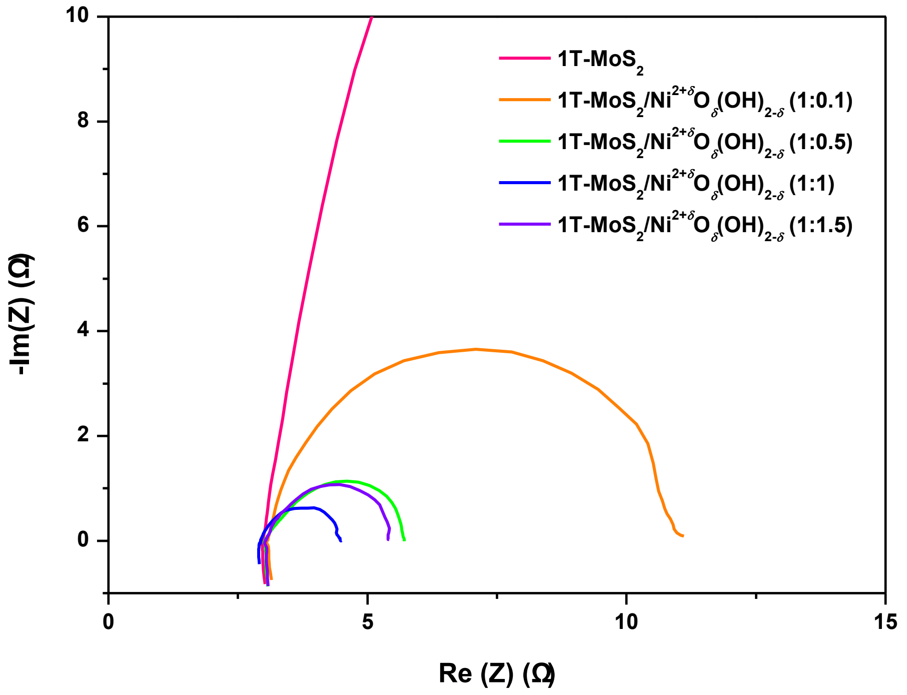

Figure S10. Electrochemical impedance spectra of the 1T-MoS_2/$Ni^{2+\delta}O_\delta(OH)_{2-\delta}$ hybrid electrodes recorded at a biased potential of -200 mV vs. RHE in 1 M KOH (mass loading: 0.4 mg cm^{-2} based on the mass of MoS_2).

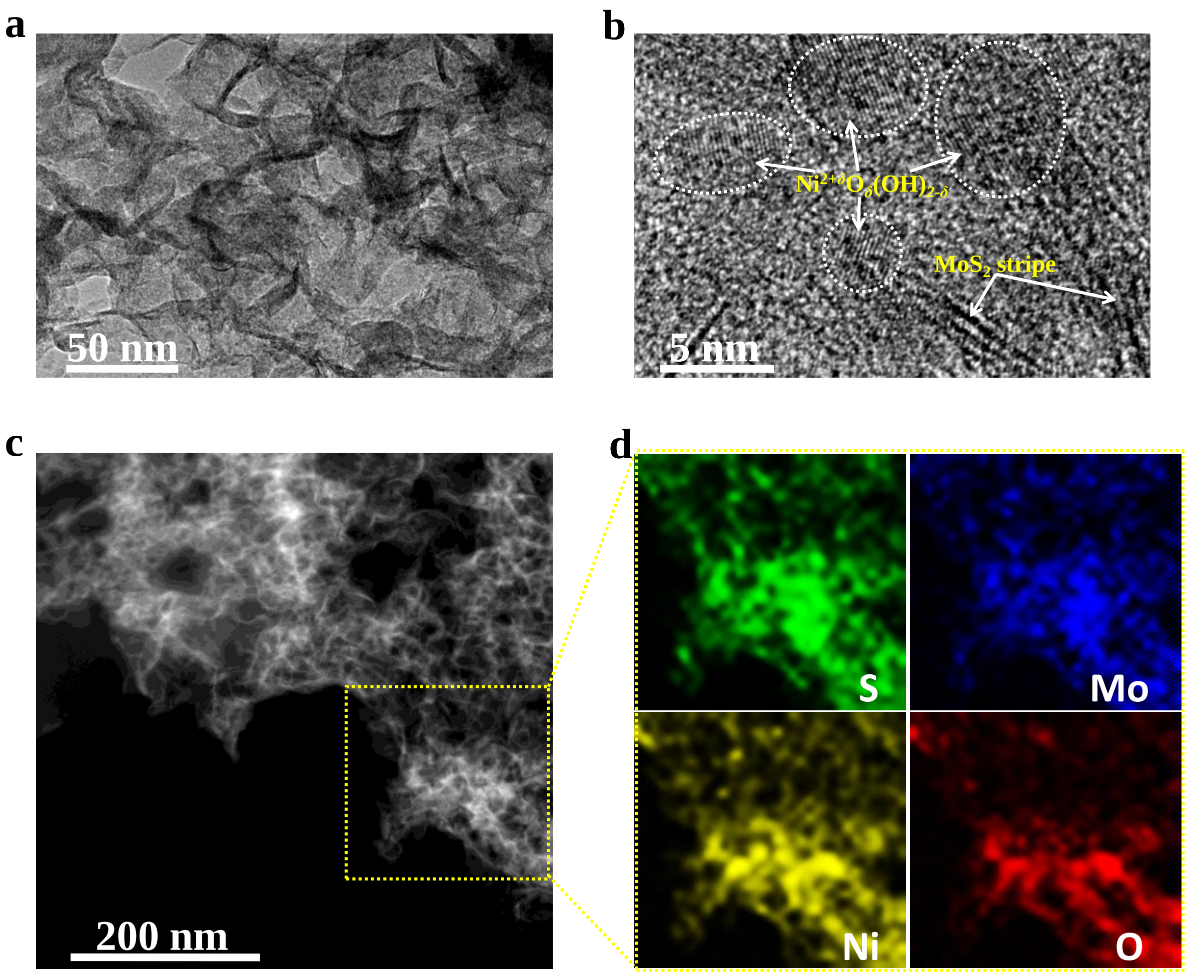

Figure S11. Morphology and composition characterizations on the post-HER 1T-MoS_2/$Ni^{2+\delta}O_{\delta}(OH)_{2-\delta}$ (1:1) hybrid (after 30 hours' chronopotentiometry test at j = -10 mA cm^{-2}). (a, b) TEM images. (c) STEM image. (d) EDS elemental mappings of S, Mo, Ni and O in the marked region in (c).

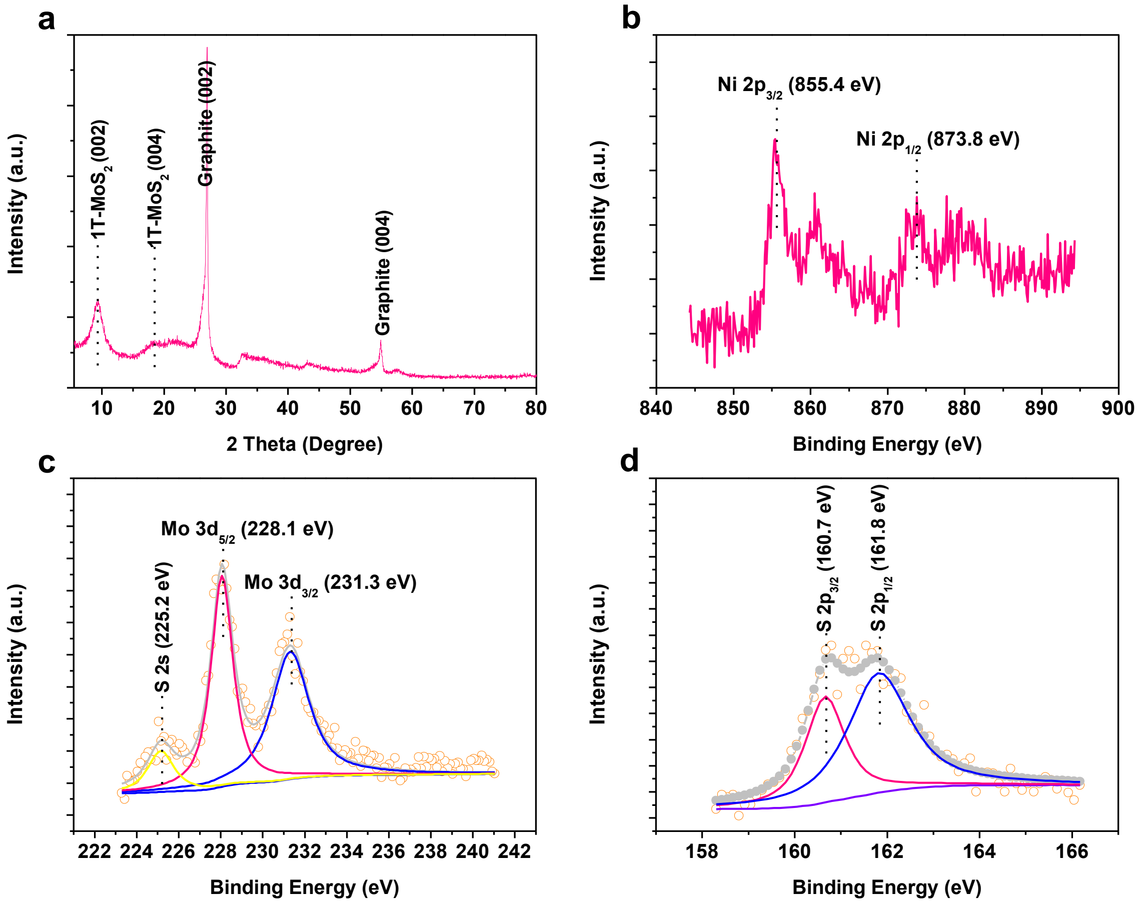

Figure S12. Structure and chemical state analyses of the post-HER 1T-MoS_2/$Ni^{2+}{}_{\delta}O_{\delta}(OH)_{2-\delta}$ (1:1)/CFP electrode (after 30 hours' chronopotentiometry test at j = -10 mA cm^{-2}). (a) XRD pattern. (b) Ni 2p, (c) Mo 3d and (d) S 2p core level XPS spectra, respectively.

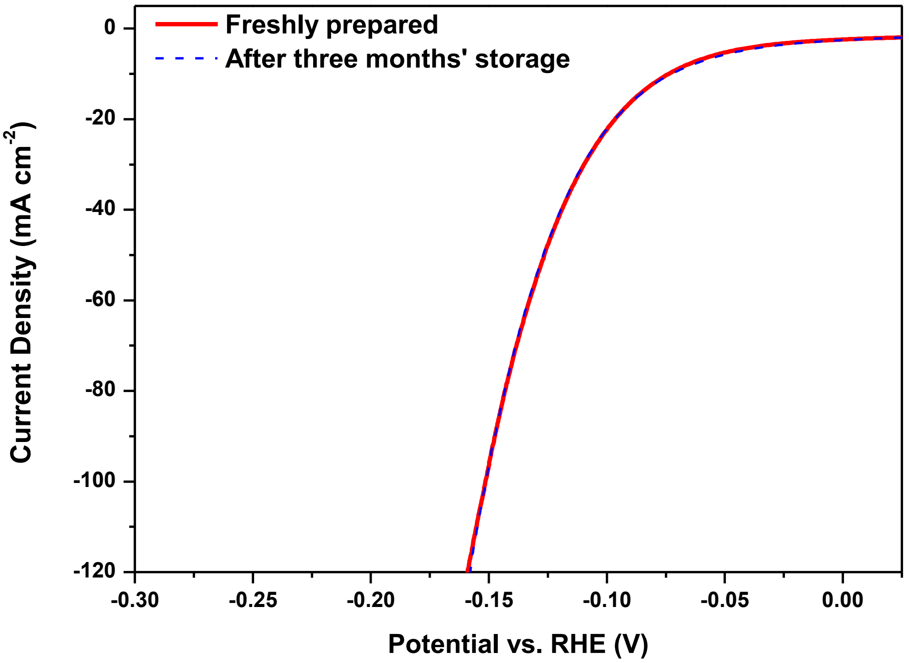

Figure S13. Polarization curves of the freshly prepared 1T-$MoS_2/Ni^{2+\delta}O_\delta(OH)_{2-\delta}$ (1:1) hybrid and that stored in ethanol over three months in 1 M KOH (mass loading: 0.4 mg cm^{-2} based on the mass of MoS_2).

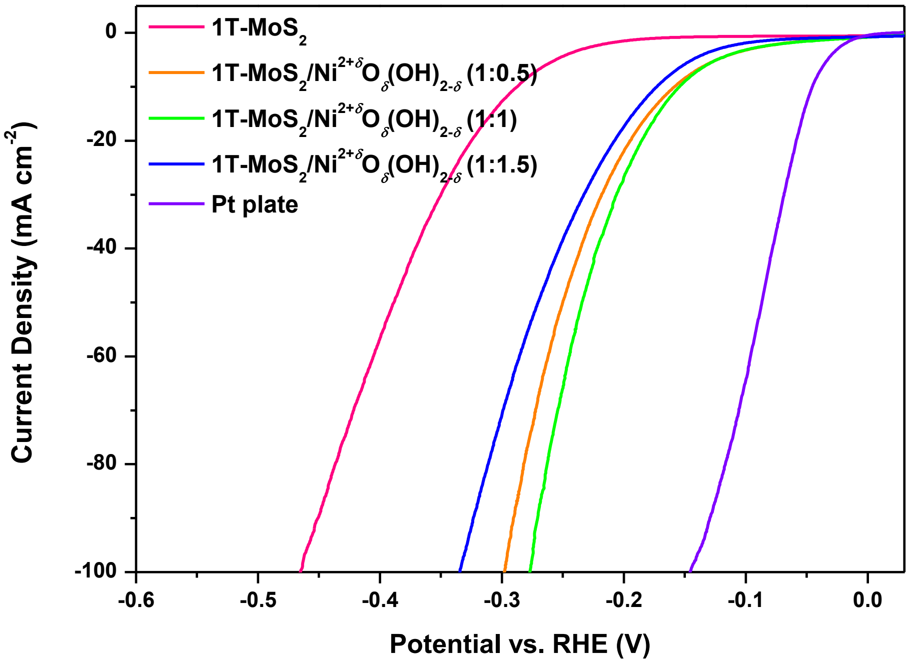

Figure S14. Polarization curves of 1T-MoS_2 and 1T-$MoS_2/Ni^{2+\delta}O_{\delta}(OH)_{2-\delta}$ hybrids for HER in neutral 1 M PBS electrolyte (mass loading: 0.4 mg cm^{-2} based on the mass of MoS_2).

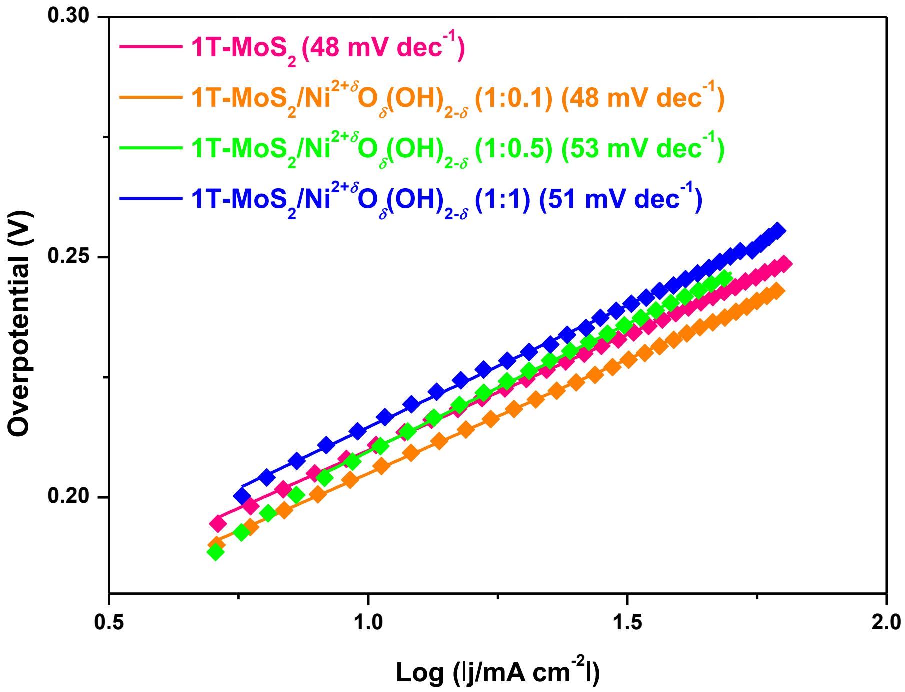

Figure S15. Tafel plots of the 1T-$MoS_2/Ni^{2+\delta}O_{\delta}(OH)_{2-\delta}$ hybrids in 0.5 M H_2SO_4 (mass loading: 0.4 mg cm^{-2} based on the mass of MoS_2).

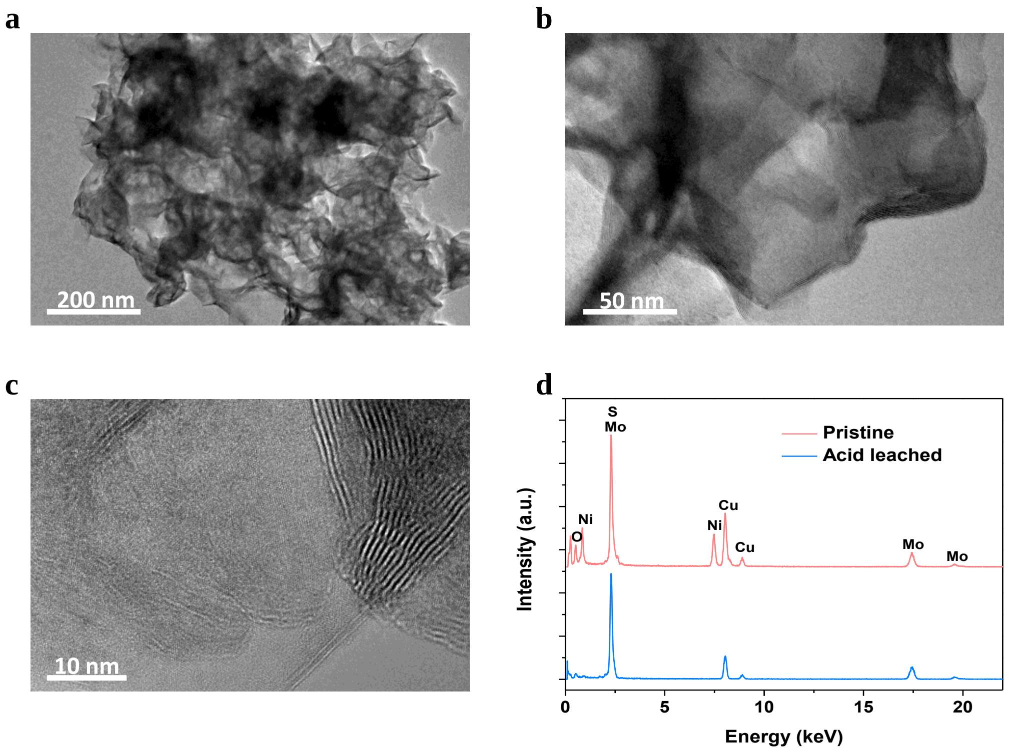

Figure S16. (a-c) TEM images of the 1T-$MoS_2/Ni^{2+}{}_{\delta}O_{\delta}(OH)_{2-\delta}$ (1:1) hybrid after thoroughly acid leaching. (d) EDX spectra of the pristine and acid leached 1T-$MoS_2/Ni^{2+}{}_{\delta}O_{\delta}(OH)_{2-\delta}$ (1:1) hybrid. The absence of any observable nanoparticles and the complete disappearance of Ni signal in the EDS of the acid leached sample demonstrate the complete remove of nickel-containing species in the hybrid after acid leaching. Acid leaching process: First, solid product collected from 20 ml of 1T-$MoS_2/Ni^{2+}{}_{\delta}O_{\delta}(OH)_{2-\delta}$ (1:1) (1 mg ml^{-1}) by centrifugation at 8000 rpm was dispersed into 30 ml 5wt% HCl aqueous solution and stirred for 10 minutes. This process was repeated with 6 times and then the collected solid product was washed with water for 3 times.

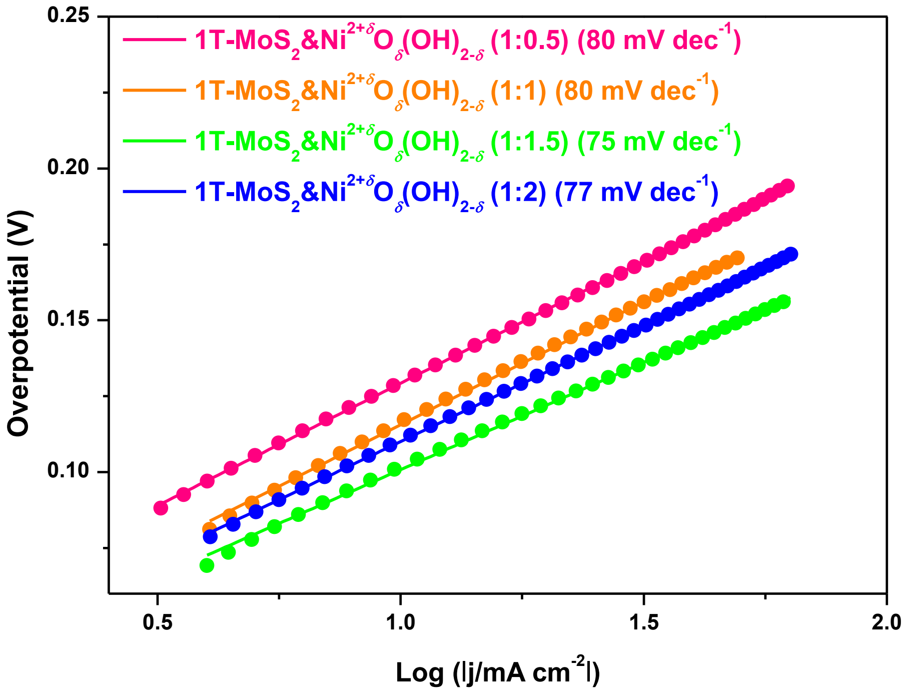

Figure S17. Tafel plots of the physical mixtures of 1T-MoS_2 and $Ni^{2+}{}_{\delta}O_{\delta}(OH)_{2-\delta}$ in 1 M KOH (mass loading: 0.4 mg cm^{-2} based on the mass of MoS_2)..

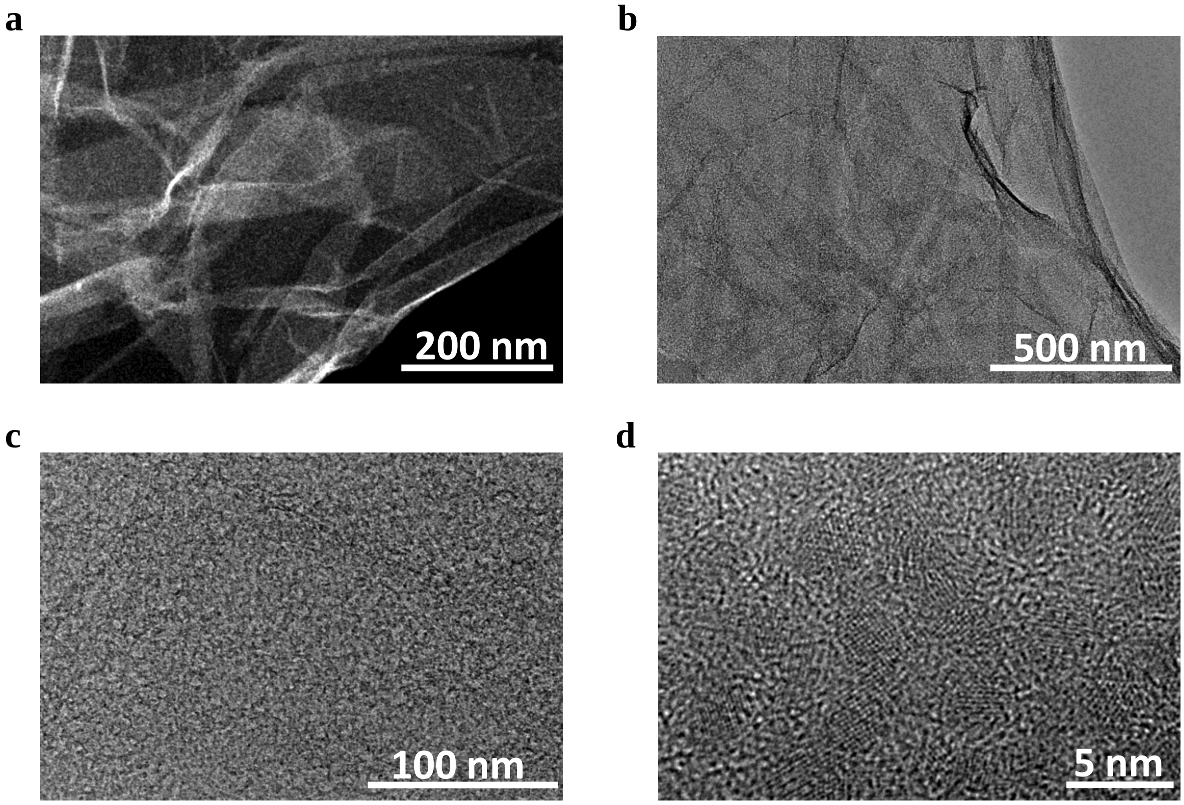

Figure S18. Electron microscopy characterizations of the GO/$Ni^{2+}{}_{\delta}O_{\delta}(OH)_{2-\delta}$ (1:1) hybrid. (a) low-magnification STEM image. (b, c) Low-magnification TEM images. (d) High-resolution TEM image. The results reveal the uniform distribution of $Ni^{2+}{}_{\delta}O_{\delta}(OH)_{2-\delta}$ nanoparticles on GO nanosheets.

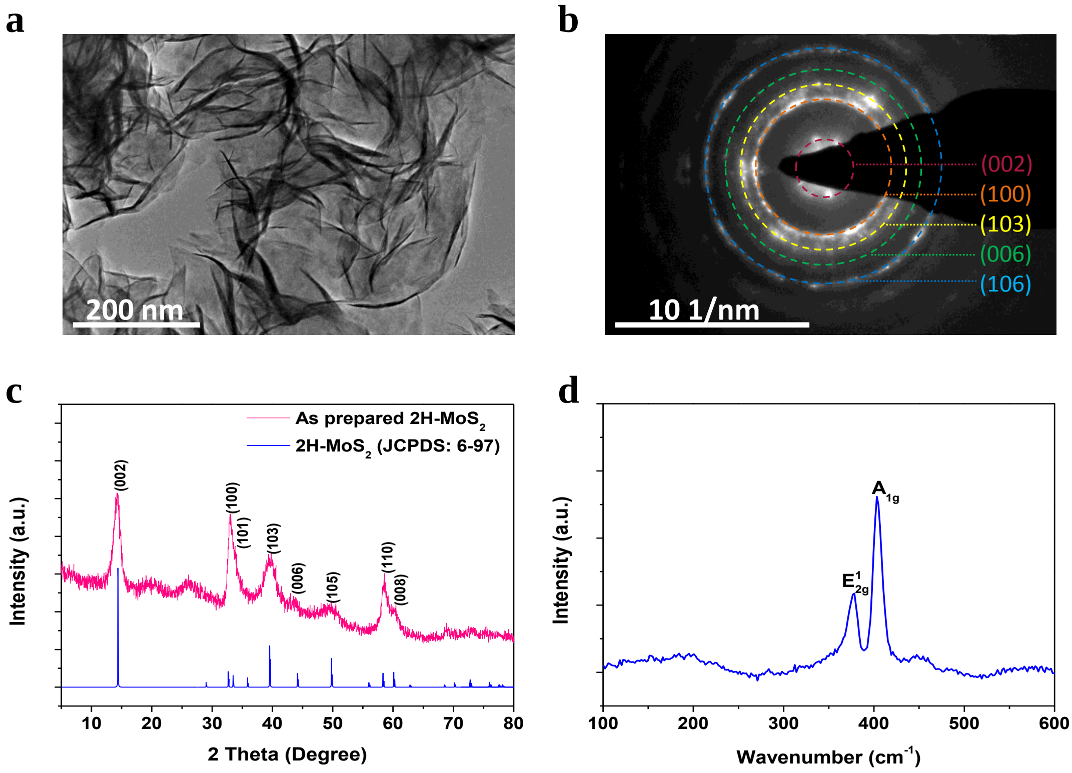

Figure S19. Morphology and structure characterizations of the prepared 2H-MoS_2. (a) TEM image, (b) SAED pattern, (c) XRD pattern and (d) Raman spectrum. All the SAED and XRD pattern are indexed with the standard diffraction pattern of 2H-MoS_2 (JCPDS No: 6-97).

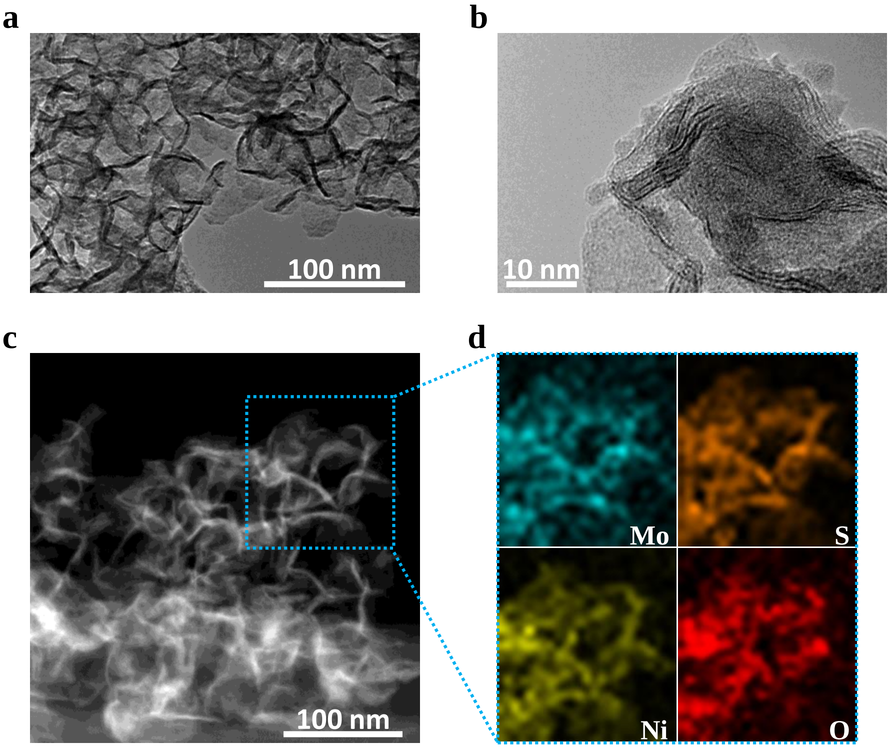

Figure S20. Morphology and structure characterizations on the 2H-MoS_2/$Ni^{2+}{}_{\delta}O_{\delta}(OH)_{2-\delta}$ (1:1) hybrid. (a, b) TEM images. (c) HAADF-STEM image. (d) Elemental mapping images of Mo, S, Ni and O.

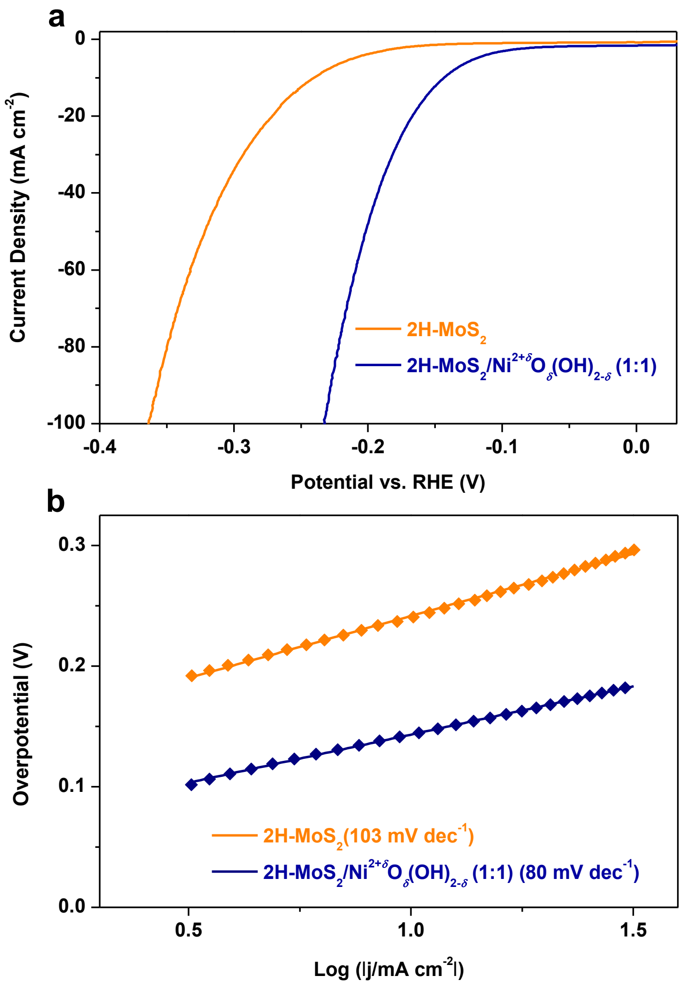

Figure S21. Comparison of HER performance between the 2H-MoS_2 and the 2H-MoS_2/$Ni^{2+\delta}O_{\delta}(OH)_{2-\delta}$ (1:1) hybrid. (a) Polarization curves. (b) Tafel plots. The measurements were conducted in 1 M KOH (mass loading: 0.4 mg cm^{-2} based on the mass of MoS_2).

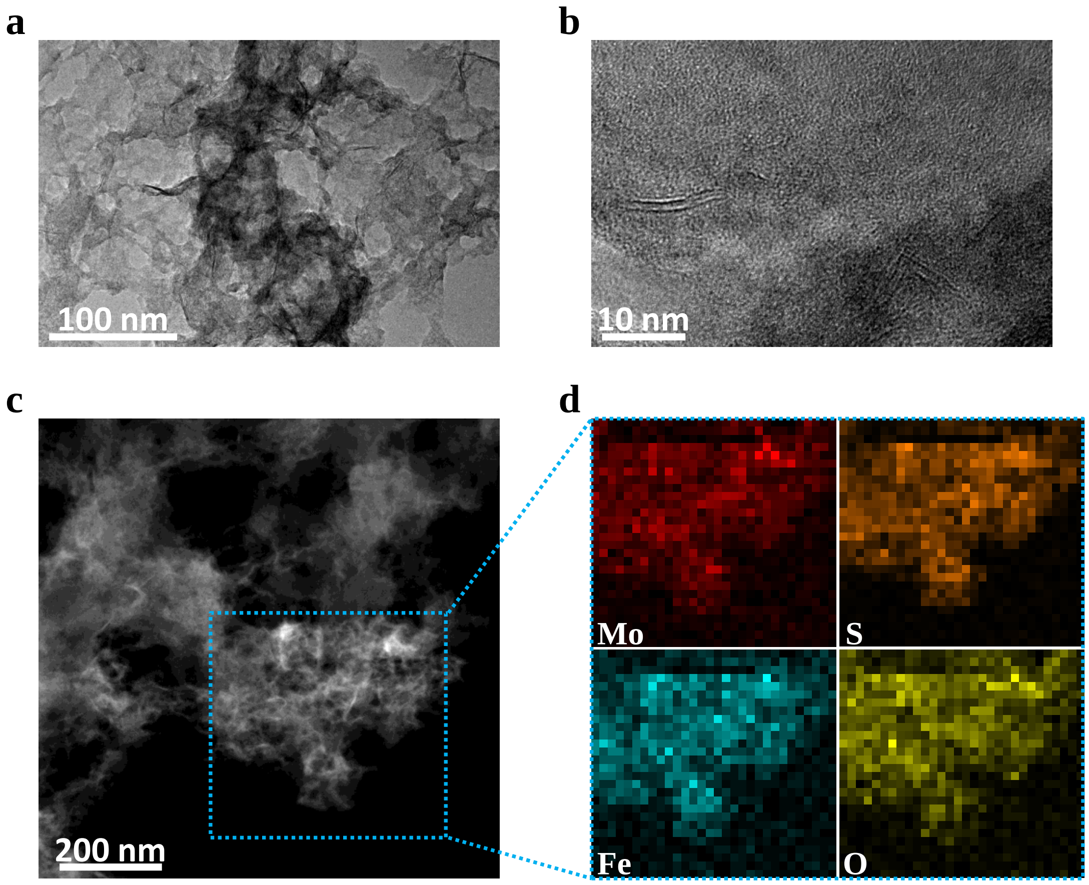

Figure S22. Morphology and structure characterizations on the 1T-$MoS_2/Fe^{2+}{}_{\delta}O_{\delta}(OH)_{2-\delta}(1:1)$ hybrid. (a, b) TEM images. (c) HAADF-STEM image. (d) Elemental mapping images of Mo, S, Fe and O.

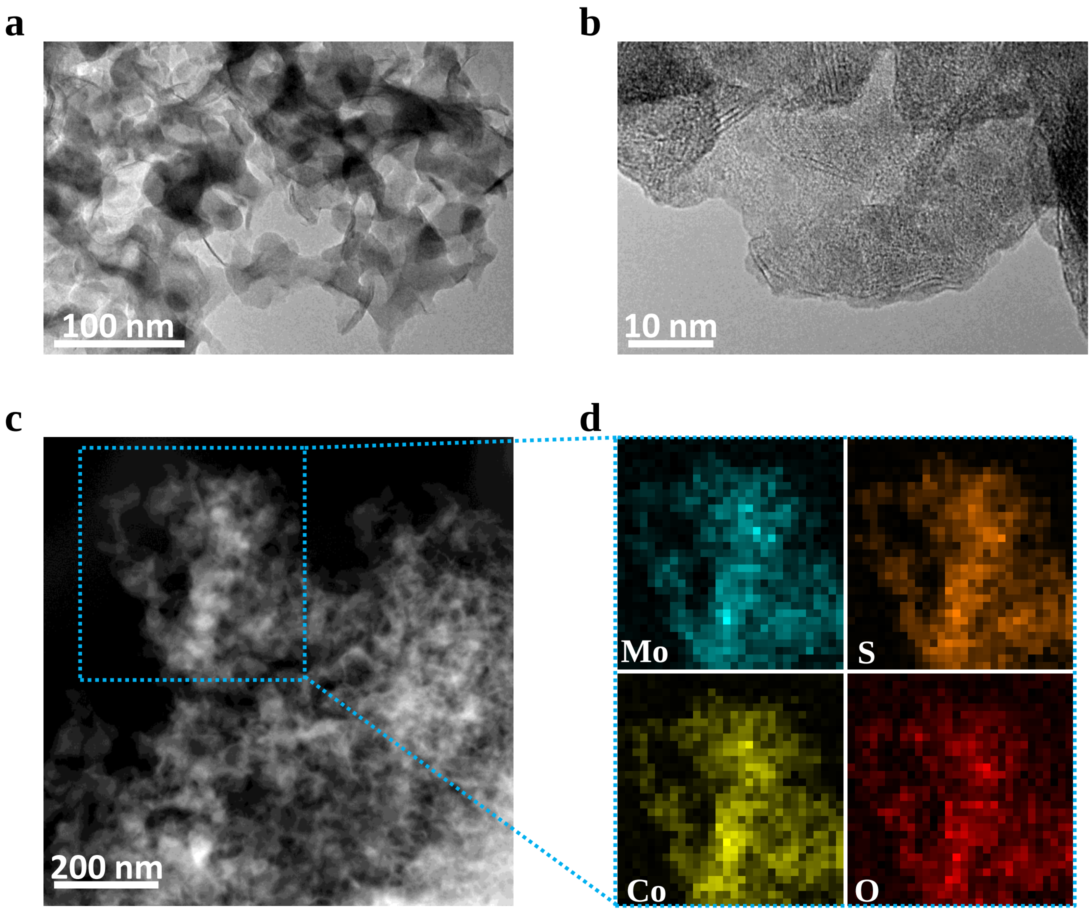

Figure S23. Morphology and structure characterizations on the 1T-$MoS_2/Co^{2+}{}_{\delta}O_{\delta}(OH)_{2-\delta}$(1:1) hybrid. (a, b) TEM images. (c) HAADF-STEM image. (d) Elemental mapping images of Mo, S, Co and O.

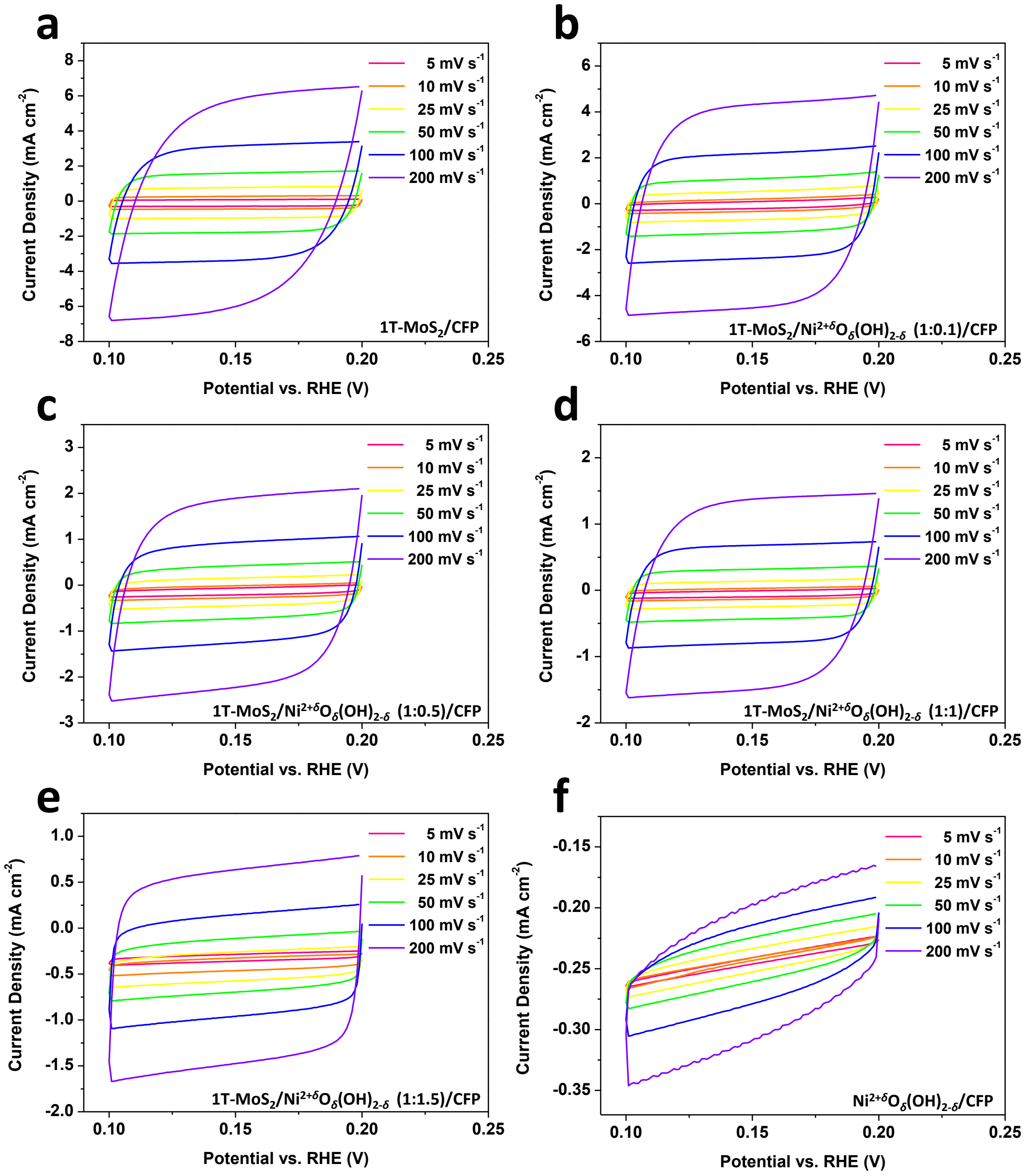

Figure S24. Cyclic voltammograms recorded at different scan rate in the non-Faradaic potential region (0.1-0.2 V vs. RHE) of various catalyst electrodes.

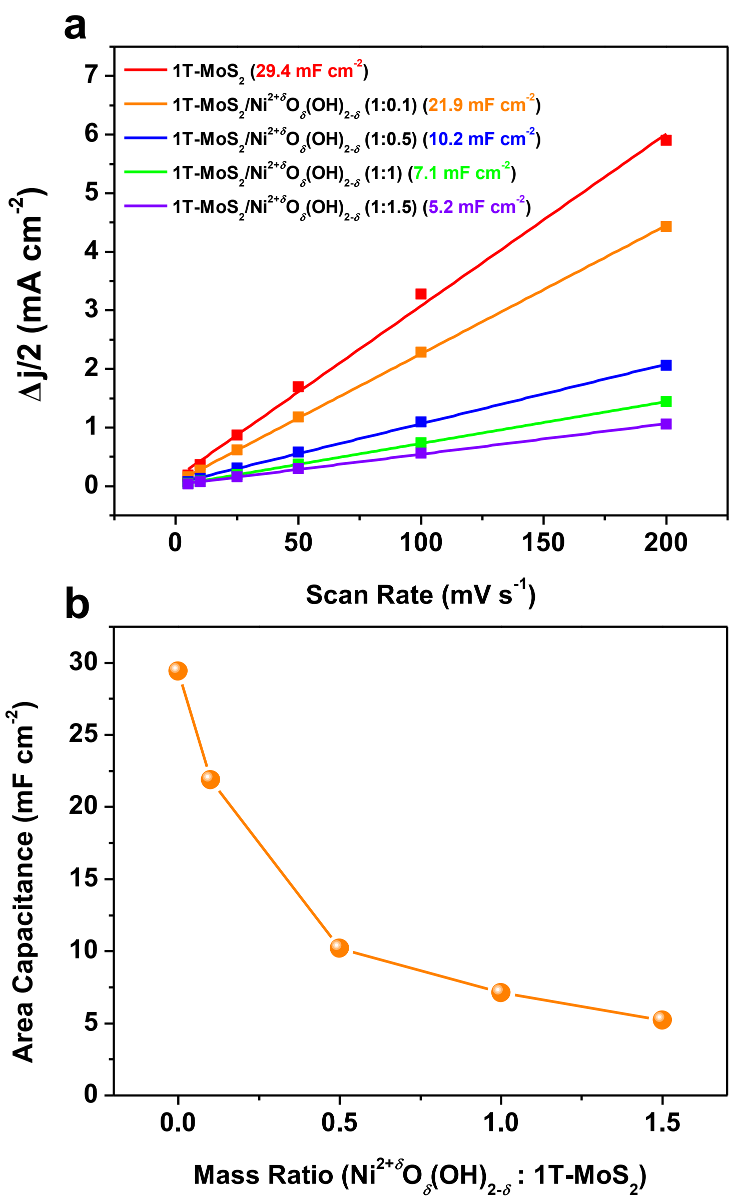

Figure S25. (a) The differences in current density ($\Delta j = j_a - j_c$) at 0.15 V vs. RHE plotted against scan rate. The fitting by a linear regression allows for the estimation of double-layer area capacitance. (b) Area capacitance plotted against the mass ratio of $Ni^{2+\delta}O_{\delta}(OH)_{2-\delta}$: 1T-MoS_2.

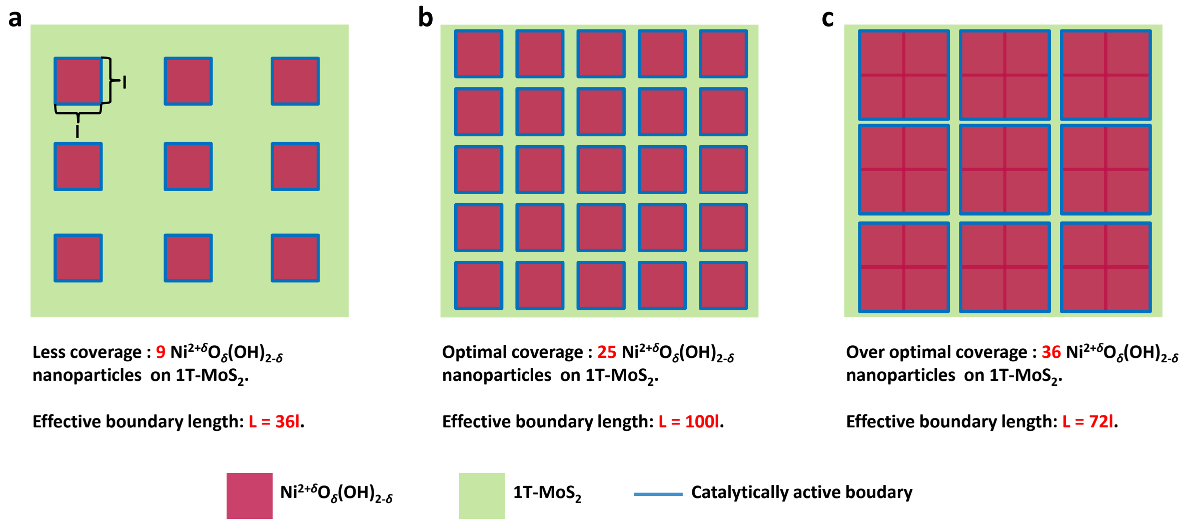

Figure S26. Simplified schematic diagram to show the effect of mass ratio of 1T-MoS_2 to $Ni^{2+}{}_{\delta}O_{\delta(OH)_{2-\delta}}$ on the overall length of the catalytically accessible boundaries in the 1T-MoS_2/$Ni^{2+}{}_{\delta}O_{\delta(OH)_{2-\delta}}$ hybrids.

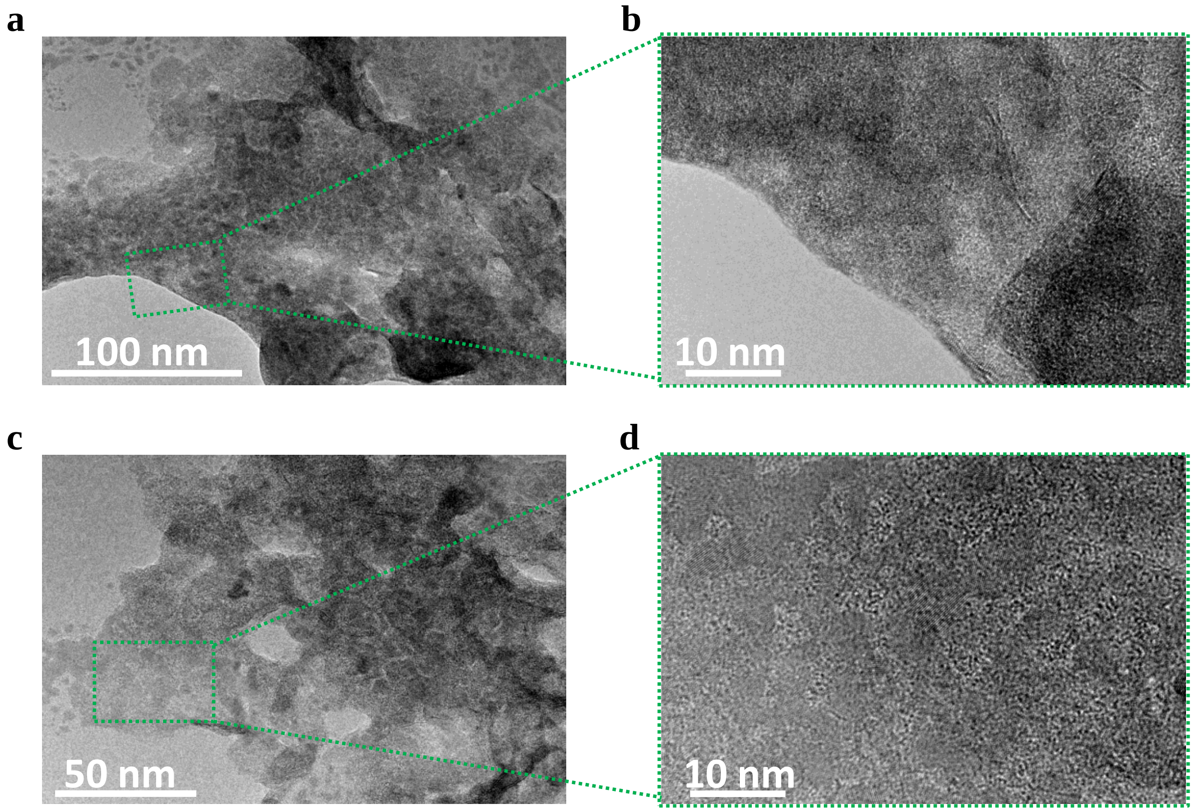

Figure S27. TEM images of the 1T-MoS_2/$Ni^{2+}{}_{\delta}O_{\delta}(OH)_{2-\delta}$ (1:1.5) hybrid. The results reveal less exposed surface of 1T-MoS_2 in the 1T-MoS_2/$Ni^{2+}{}_{\delta}O_{\delta}(OH)_{2-\delta}$ (1:1.5) hybrid than that in the 1T-MoS_2/$Ni^{2+}{}_{\delta}O_{\delta}(OH)_{2-\delta}$ (1:1) hybrid.

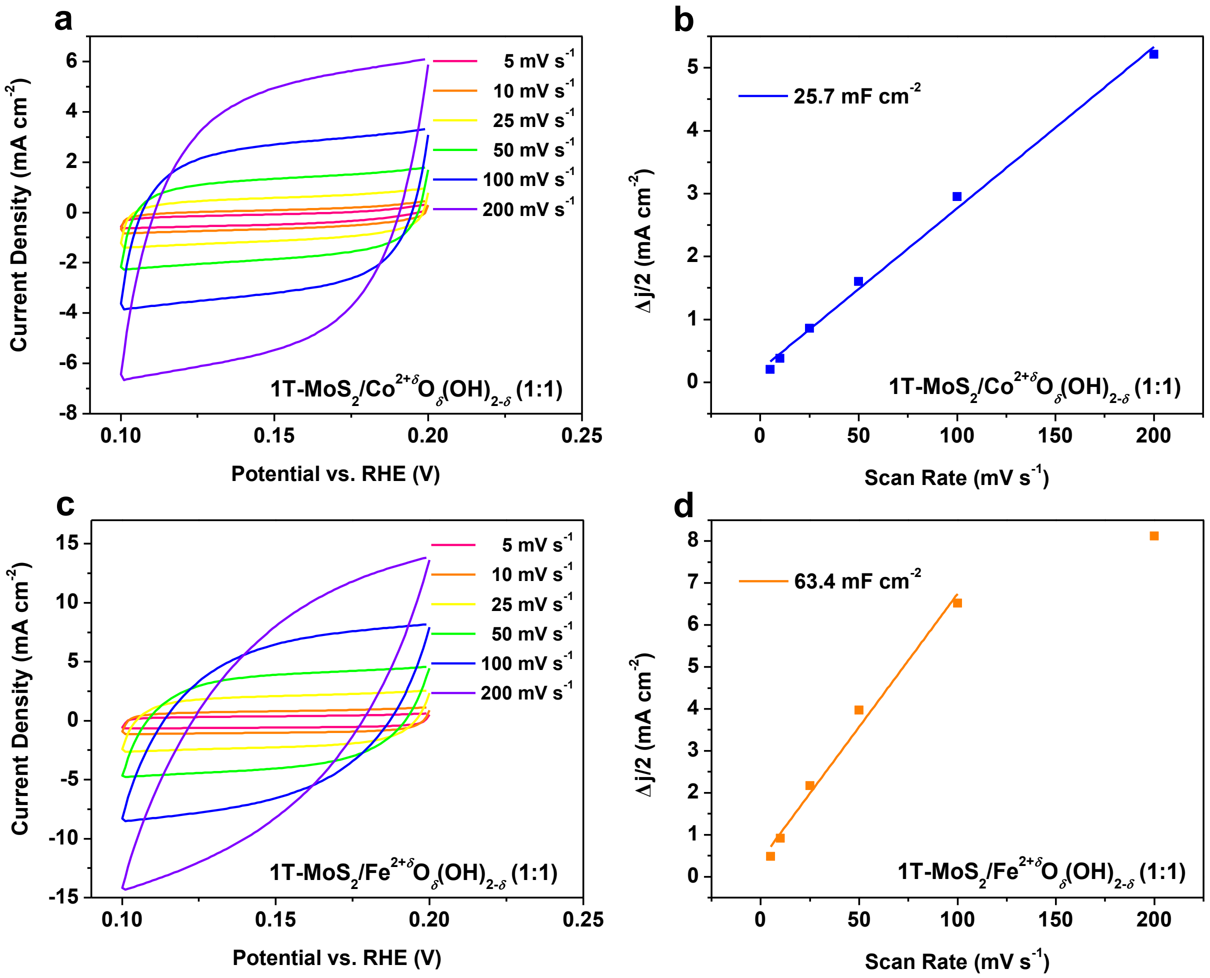

Figure S28. Cyclic voltammograms recorded at different scan rate in the non-Faradaic potential region (0.1-0.2 V vs. RHE) of (a) 1T-MoS_2/$Co^{2+}{}_\delta O_{\delta(OH)_{2-\delta}}$ $_{(1:1)}$ hybrid and (b) 1T-MoS_2/$Fe^{2+}{}_\delta O_{\delta(OH)_{2-\delta}}$ $_{(1:1)}$ hybrid. (c, d) The fitting by a linear regression allows for the estimation of their double-layer area capacitances.

Table S1. A survey of the HER performances of non-precious metal based electrocatalysts in alkaline electrolytes from recent literatures.

Catalyst	Mass Loading (mg cm^{-2})	η (mV) @ j=10 mA cm^{-2} (Electrolyte)	Tafel Slope (mV dec^{-1})	Reference (Publication year)
1T-MoS_2/$Ni^{2+\delta}O_\delta(OH)_{2-\delta}$ (1:1)	**0.8**	**73** (1 M KOH)	**77**	**This work**
$CoMoS_x$	0.05	158 (5 mA cm^{-2}) (0.1 M KOH)	N/A	*Nat. Mater.* **15**, 197-203 (2016)
MoC_x nano-octahedrons	0.8	151 (1 M KOH)	59	*Nat. Commun.* **6**, 6512 (2015).
CoN_x/C	2.0	170 (1 M KOH)	75	*Nat. Commun.*, **6**, 7992 (2015).
Ni/NiO-CNT	0.28	~86 (1 M KOH)	82	*Nat. Commun.* **5**, 4695 (2014).
Li-$NiFeO_x$	1.6	88 (1 M KOH)	150	*Nat. Commun.*, **6**, 7261 (2015).
Co-NG	0.28	~270 (1 M NaOH)	N/A	*Nat. Commun.*, **6**, 8668 (2016).
C_3N_4@NG	0.1	> 600 (0.1 M KOH)	N/A	*Nat. Commun.*, **5**, 3783 (2014).
Ni-MoS_2	0.89	98 (1 M KOH)	60	*Energy Environ. Sci.* **9**, 2789-2793 (2016).
CoP nanowire array	0.9	209 (1 M KOH)	129	*J. Am. Chem. Soc.* **136**, 7587-7590 (2014).
Bulky MoP	0.86	~130 (1 M KOH)	48	*Energy Environ. Sci.* **7**, 2624-2629 (2014).

MoB	2.3	220 (1 M KOH)	59	*Angew. Chem. Int. Ed.* **53**, 6710-6714 (2014).
Mo_2C	0.8	190 (1 M KOH)	54	
NiSe nanowire array	2.8	96 (1 M KOH)	120	*Angew. Chem. Int. Ed.* **54**, 9351-9355 (2015).
$Ni(OH)_2/MoS_2$	~4.8	80 (1 M KOH)	60	*Nano Energy* **37**, 74-80. (2017)
Co-P film	~2.7	94 (1 M KOH)	42	*Angew. Chem. Int. Ed.* **54**, 6251-6254. (2015)
h-NiS_x	142.2	60 (1 M KOH)	99	*Adv. Energy Mater.* **6**, 1502333. (2016)
Mo_2C@C S-800	~0.9	47 (1 M KOH)	71	*ACS Nano* **10**, 8851-8860. (2016)
np-$(Co_{0.52}Fe_{0.48})_2P$	Unknown	79 (1 M KOH)	40	*Energy Environ. Sci.* **9**, 2257-2261. (2016)
porous MoO_2	~2.9	27 (1 M KOH)	41	*Adv. Mater.* **28**, 3785-3790. (2016)
Fe-Doped CoP Nanoarray	~1.03	78 (1 M KOH)	75	*Adv. Mater.* **29**, 1602441. (2017)
$NiCo_2P_x$ nanowire array	5.9	58 (1 M KOH)	34.3	*Adv. Mater.* **29**, 1605502. (2017)
$Ni_{0.89}Co_{0.11}Se_2$ MNSN	2.16	85 (1 M KOH)	52	*Adv. Mater.* **29**, 1606521. (2017)
2.5H-PHNCMs	0.196	70 (1 M KOH)	38.1	*Nat. Commun.* **8**, 15377. (2017)
$MoNi_4/MoO_2$@Ni	~43.4	15 (1 M KOH)	30	*Nat. Commun.* **8**, 15437. (2017)

Table S2. A survey of the HER performances of non-precious metal based electrocatalysts in neutral electrolytes from recent literatures.

Catalyst	Mass Loading (mg cm^{-2})	η vs. RHE (mV) @ j=10 mA cm^{-2} (Electrolyte)	Tafel Slope (mV dec^{-1})	Reference (Publication year)
1T-$MoS_2/Ni^{2+\delta}O_{\delta}(OH)_{2-\delta}$ (1:1)	**0.8**	**153** (1 M PBS)	**106**	**This work**
CoN_x/C	2.0	247 (1 M PBS)	N/A	*Nat. Commun.* **6**, 7992 (2015).
Cu_2MoS_4/C	0.04	750 (0.1 M PBS)	~100	*Energy Environ. Sci.* **5**, 8912 (2012).
Co NPs@N-CNTs	0.28	540 (0.1 M PBS)	N/A	*Angew. Chem. Int. Ed.* 53, 4372-4376 (2014).
Ni-Mo-S/C (1:1)	0.52	200 (0.5 M PBS)	85.3	*Sci. Adv.* **1**, e1500259 (2015).
Ni_3S_2/NF	1.6	170 (Unknown)	N/A	*J. Am. Chem. Soc.* **137**, 14023 (2015).
CoP nanorod array	0.92	106 (1 M PBS)	93	*J. Am. Chem. Soc.* **136**, 7587 (2014).
Amorphous Co-S film	N/A	160 (1 M PBS)	93	*J. Am. Chem. Soc.* **135**, 17699 (2013).
